# Obesity-associated inflammation countered by a Mediterranean diet: the role of gut-derived metabolites

**DOI:** 10.3389/fnut.2024.1392666

**Published:** 2024-06-24

**Authors:** Melanie Florkowski, Esther Abiona, Karen M. Frank, Allison L. Brichacek

**Affiliations:** Department of Laboratory Medicine, National Institutes of Health Clinical Center, Bethesda, MD, United States

**Keywords:** Mediterranean, obesity, microbiome, metabolites, inflammation, short-chain fatty acids, trimethylamine N-oxide, bile acids

## Abstract

The prevalence of obesity has increased dramatically worldwide and has become a critical public health priority. Obesity is associated with many co-morbid conditions, including hypertension, diabetes, and cardiovascular disease. Although the physiology of obesity is complex, a healthy diet and sufficient exercise are two elements known to be critical to combating this condition. Years of research on the Mediterranean diet, which is high in fresh fruits and vegetables, nuts, fish, and olive oil, have demonstrated a reduction in numerous non-communicable chronic diseases associated with this diet. There is strong evidence to support an anti-inflammatory effect of the diet, and inflammation is a key driver of obesity. Changes in diet alter the gut microbiota which are intricately intertwined with human physiology, as gut microbiota-derived metabolites play a key role in biological pathways throughout the body. This review will summarize recent published studies that examine the potential role of gut metabolites, including short-chain fatty acids, bile acids, trimethylamine-N-oxide, and lipopolysaccharide, in modulating inflammation after consumption of a Mediterranean-like diet. These metabolites modulate pathways of inflammation through the NOD-like receptor family pyrin domain-containing 3 (NLRP3) inflammasome, toll-like receptor 4 signaling, and macrophage driven effects in adipocytes, among other mechanisms.

## Graphical Abstract

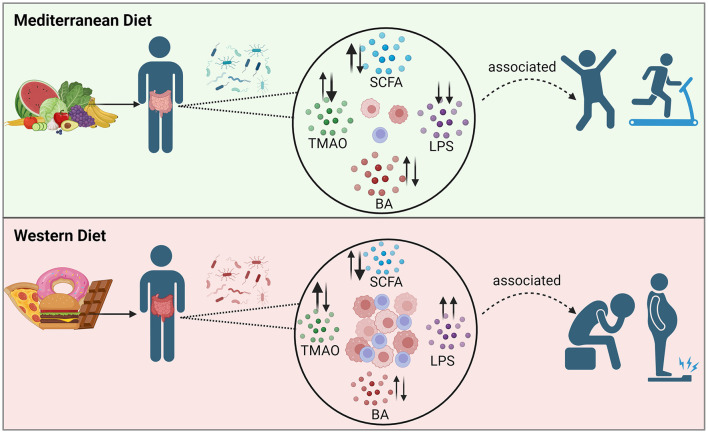

## 1 Introduction

The World Health Organization (WHO) estimates that over 1 billion individuals worldwide now grapple with overweight/obesity^1^. Obesity is associated with numerous comorbid conditions, notably non-communicable diseases including hypertension, type 2 diabetes (T2D), and cardiovascular disease (CVD) which contributed to a staggering five million deaths globally in 2019 (1–3). These comorbid conditions present a significant health burden in individuals with overweight/obesity and make combating obesity a public health priority. Complex factors influence the prevalence of obesity, including genetics, physical activity levels, dietary pattern, caloric intake, medical conditions and their treatments, socioeconomic status, sleep habits, stress, and environmental chemicals (4). Inflammation, particularly chronic low-grade inflammation, has been implicated in numerous non-communicable diseases including obesity, metabolic syndrome, T2D, CVDs, and certain cancers (5). Research suggests that inflammation is a key driver of obesity (6) and that excess adipose tissue and dysfunctional adipocytes contribute to increased inflammation (7). Obese individuals have higher circulating inflammatory markers than lean individuals, and those markers are lowered following weight loss (8). Increasing inflammation in rodent models induces weight gain (6), and treatment with the anti-inflammatory cytokine interleukin (IL)-10 alleviated high-fat diet (HFD)-induced obesity (9). The aim of this review is to discuss recent studies that examine the influence of a Mediterranean diet (MedDiet) on inflammation and obesity. Specifically, we are interested in the observed effects of MedDiet adherence on gut-derived metabolites and their role in the physiology of obesity. There are multiple pieces of evidence required to connect specific dietary elements to conditions such as obesity and heart disease: (1) how digested food affects the gut microbiota composition, (2) which/how specific gut microbes in the host environment affect which gut metabolites that are present and in what quantities, (3) which/how gut metabolites influence cellular functions and biological pathways, and (4) which pathways are part of the physiology of healthy or diseased states. There have been a number of excellent reviews on some of the topics within this review, such as reviews on the MedDiet and inflammation (5) the MedDiet and the gut microbiome (10), or the role of some of the gut metabolites in obesity (11–14). We present a review that highlights the most recent literature and discusses all these topics: the MedDiet, obesity, inflammation, and gut metabolites, with a focus on the updates for four of the gut-derived metabolites that have been the focus of multiple recent investigations. Each individual scientific study may focus on only one of the four elements. In this review, we introduce the associations between obesity and inflammation, then we focus in more detail on the evidence for biological roles of specific metabolites. We focus on the most recent research results related to the pathways that include four gut metabolites (short-chain fatty acids, bile acids, trimethylamine N-oxide, and lipopolysaccharide) and discuss gaps in our understanding.

## 2 Method

For this narrative review, most articles included were chosen from searches in PubMed and Google Scholar. The online searches were conducted from September 2023–May 2024 using the keywords: Mediterranean, diet, food, Western, microbiome, bacteria, microbiota, obesity, obese, short-chain fatty acid (SCFA), lipopolysaccharide (LPS), bile acid (BA), trimethylamine N-oxide (TMAO), inflammation, inflammatory, immune, immunity, gut, metabolites, metabolomics, pathophysiology, pathway, chronic disease, and combinations thereof. Additional relevant publications were found in the citations of the articles found in our literature search. We included original research articles, reviews, meta-analyses, and clinical trials. Publications were restricted to the English language, selected on a discretionary basis by a consensus of the four authors, and we prioritized articles published within the last 4 years, though other older relevant articles were included. We focused on a subset of human studies of recently published original research reports that investigate the association between MedDiet adherence, inflammation, obesity, and gut metabolites, but also included studies in animal models that investigated biological pathways relevant to gut metabolites and inflammation.

## 3 Dietary contribution to inflammation and obesity

The escalating health burden of obesity has prompted research into its causes and possible preventive measures, particularly in modifiable lifestyle factors such as diet. In addition to energy intake, diet may also mediate other determinates of obesity such as inflammation, and there are multiple studies examining the role of nutrition in low-grade inflammation. Unraveling causality and defining pathways that connect nutrition and inflammation has proven very challenging due to the multifaceted nature of inflammatory pathways (15, 16). Pathways identified as important in inflammation, as related to diet and obesity, include the NLRP3 inflammasome, macrophage-mediated chronic low-grade inflammation in adipose tissue, and the toll-like receptor 4 (TLR4) signaling pathway that is activated by saturated fatty acids. C-reactive protein (CRP), adipocyte-derived metabolites, and inflammatory cytokines [such as tumor necrosis factor alpha (TNF-α), IL-1β, and IL-6] have been shown to play a role in inflammation associated with obesity, and in the development of insulin resistance (7, 15, 17–20). Indices like the Dietary Inflammatory Index (DII) have been developed to assess the inflammatory potential of a diet (21). A large study of more than 27,000 individuals over a period of about a decade found an association of overall obesity and abdominal obesity with a poor quality, pro-inflammatory diet. The authors used three indices, the Alternative Healthy Eating Index (AHEI), DII, and MedDiet Score, and found the AHEI to provide the best assessment of obesogenic potential of a diet, though the three indices have generally similar items in their assessment (22).

Several excellent reviews have been published that outline the connections between obesity, inflammation, and immunity. The review by May and den Hartigh focused on the impact of SCFAs on adipose tissue metabolism (23). A comprehensive review of the association of diet and gastrointestinal immunity itemized specific physiological effects associated with particular dietary macromolecules (24). A review of recent advances in our understanding of intestinal immunometabolism and microbiology provided a description of physiological differences between lean and obese states (25). Grosso et al. effectively summarized the proposed role of specific dietary elements, including macronutrients and phytochemicals, in the regulation of inflammation and immunity as relates to obesity (15). A review of ten meta-analyses summarized the evidence connecting the MedDiet with reduced dyslipidemia and decreased inflammatory mediators through modulation of the gut microbiota (10). Given the extensive data associating obesity and inflammation, combined with the data associating dietary changes with inflammation, dietary changes are justifiably proposed as one critical component of the treatment for obesity.

### 3.1 MedDiet

The MedDiet, originating from the traditional practices of people in the Mediterranean basin, has captured researchers' attention due to its reported health benefits, and is included as a healthy dietary pattern in the 2020–2025 Dietary Guidelines for Americans (26–28). The MedDiet promotes daily consumption of whole grains, nuts, vegetables, and fruit, with olive oil as the primary fat, moderate intake of fish, poultry, and wine, and rare intake of red meat and sweets (26, 29, 30). Anti-inflammatory effects have been attributed to multiple specific elements of the MedDiet, investigated alone in controlled studies, including olive oil, nuts, fatty fish, legumes, fruit, vegetables, and a reduction of red meat and refined foods (5). Although there is a general consensus regarding the characteristics of the MedDiet, criteria for calculating a “MedDiet score” vary considerably between studies (22, 29, 31). Regardless of the details of the MedDiet score, there is an abundance of data on the benefits of a MedDiet. A systematic review of 84 studies concluded that there is strong evidence to support an association of the MedDiet with fewer chronic diseases, including neurological diseases, CVD, cancer, T2D, liver disease, and renal disease. The MedDiet was also associated with reduced obesity-related metabolic features, inflammation, and lower mortality (31).

The Western diet, in contrast to the MedDiet, is a dietary pattern prevalent across many industrialized nations. Key components of the Western diet include high consumption of refined grains, red meat, and sugar sweetened beverages, which are associated with weight gain and obesity risk (10, 15, 22, 24, 32, 33). The Western diet can also include 50% or more of the calories from foods that are classified as ultra-processed, meaning they contain formulations of ingredients assembled in industrial processes as opposed to whole foods. Studies have associated ultra-processed foods (UPF) with low-grade inflammation and multiple chronic diseases (32, 34). Our review is not primarily focused on UPFs; however, individuals following a MedDiet, or other similar healthy dietary patterns, tend to consume fewer UPFs and would be spared the inflammatory response, and consequence of the inflammation, that may be associated with them.

### 3.2 Effects of a MedDiet vs. a Western diet on obesity and inflammation

The benefits of the MedDiet have been evaluated in many observational and intervention studies of obesity and its comorbidities, suggesting that the MedDiet can ameliorate obesity across various populations. Although there are numerous investigations of dietary patterns, or specific dietary components, and the health consequences, we will focus on a subset of studies: recently published original research reports that investigate the association between MedDiet adherence, inflammation, and obesity.

#### 3.2.1 Observational studies of the effect of the MedDiet on obesity and inflammation

The results of recent observational studies provide supporting data for the association of the MedDiet with weight loss and reduced inflammation. Dietary intervention studies have shown that the MedDiet, with or without caloric restriction, may induce weight loss in individuals with overweight and obesity (35–37). A study of self-selected diets by individuals with obesity found the MedDiet resulted in an average weight loss of 2.8 kg after 12 months. The weight loss induced by the other diets evaluated, Paleo and intermittent fasting, showed similar results to the MedDiet in this study (38).

Beyond examination of weight changes, studies have reported changes in inflammatory cytokines and a decrease in comorbidities in association with a MedDiet, even without weight loss. A multi-year study of over 39,000 individuals who were included in the Melbourne Collaborative Cohort found an association between the development of T2D and a higher DII, as well as a lower AHEI, but they did not find an association with the MedDiet score in this study (39). A study of 238 individuals who had non-alcoholic fatty liver disease, now called metabolic dysfunction-associated steatotic liver disease (MASLD) (40) showed that adherence to a MedDiet, as assessed by questionnaire, correlated with lower oxidative stress and inflammation (41). Monitoring of 612 subjects during a year-long study found an association between adherence to a MedDiet and lower inflammatory markers, CRP and IL-17. Additionally, changes in the gut microbiome seen with MedDiet adherence correlated with lower frailty, improved cognition, and reduced inflammation (42). In the observational study of 307 male participants as part of the Health Professionals Follow-up study that involved broad examination of sequence data, food logs, and blood biomarkers, long-term adherence to the MedDiet was associated with a change in the gut microbiome and their associated metabolic pathways, including SCFAs, secondary BA production, and fiber metabolism. They did not find an association of the MedDiet with the abundance of *Prevotella copri*, but they did find an association between the presence of *P. copri* with reduced risk of CVD, allowing for hypotheses of the pathways of this species that contribute to the observed phenotype (43).

A study of 1,040 individuals, as a subset of the Hellenic National Nutrition and Health Survey, found a significant association between adherence to the MedDiet, lower weight, and reduced hypertension (44). A cross-sectional study of 65 individuals examined the association of diet and inflammation using food diaries, hyperinsulinemic-euglycemic clamps, intravenous glucose tolerance test, dual-energy X-ray absorptiometry, cytokine levels, and adipokine levels. Adherence to a MedDiet was associated with greater insulin sensitivity and decreased inflammatory markers in adults with overweight/obesity (45). One study showed that women with obesity with higher adherence to the MedDiet had lower incidence of MASLD (46). High adherence to the MedDiet was observed to lower the risk of developing an unhealthy metabolic phenotype in individuals with and without obesity (47). Women with obesity and polycystic ovary syndrome who had higher adherence to the MedDiet also had lower cardiometabolic risk factors, including reduced levels of CRP, insulin resistance, and fatty liver index (48). Whole grain consumption, a component of the MedDiet, is also associated with decreased inflammation, in contrast to consumption of refined grains, in part due to its increased amount of dietary fiber (49). The MedDiet also discourages the consumption of red meat which has been consistently associated with inflammation, in favor of poultry or fish, the latter of which are high in omega-3 polyunsaturated fatty acids (50–52).

One strength of observational studies is that they can be quite large with thousands of participants, creating the potential for a statistically very well-powered study. Limitations of observational studies include the uncontrolled variables of each study that are outside of the diet being examined, such as physical activity, sleep habits, stress from injury or other medical conditions, all of which can affect the inflammatory state of the participants. The level of detail of the diets is less than can be obtained in a controlled trial for which food is provided. The range of what is considered a MedDiet might include those scoring anywhere from 10 to 17, out of 17 total points that describe a “fully-compliant” MedDiet on a PREDIMED score for example, so the food consumed by all of the participants in the “MedDiet” group could be quite variable, affecting the results of one study as compared to another. An additional limitation of these studies is that they cannot directly examine specific biological pathways. Despite the limitations of these studies, the strength of the collective evidence supports the role of the MedDiet in reducing obesity-associated inflammation and comorbidities.

#### 3.2.2 Randomized controlled trials of the effect of the MedDiet on obesity and inflammation

Observational studies frequently include large cohorts for statistical power but randomized controlled trials (RCTs) add a layer of rigor and control to the results, moving us closer to determining the cause of the investigated effect. A randomized dietary intervention study of individuals with obesity and features of metabolic syndrome compared 128 genes expressed in abdominal subcutaneous adipose tissue for those on a Nordic diet, which is a Nordic alternative to the MedDiet, and those on a control diet. The authors concluded that the Nordic diet was associated with a decrease in inflammatory gene expression (53). A randomized controlled trial of 82 subjects with overweight/obesity comparing the MedDiet to a control diet demonstrated significant changes in the endocannabinoid system, along with an increase in *Akkermansia muciniphila* on the MedDiet. The change in the oleoylethanolamide/palmitoylethanolamide (OEA/PEA) endocannabinoid ratio following the MedDiet also diminished the homeostatic model assessment of insulin resistance index and decreased serum high-sensitive CRP, a measure of systemic inflammation. Their results support a role for the MedDiet in ameliorating insulin sensitivity and inflammation (54). A randomized controlled trial involving 28 adults with quiescent ulcerative colitis found that a MedDiet reduced levels of fecal calprotectin, a measure of intestinal inflammation (55). Higher adherence to the MedDiet is associated with lower inflammatory biomarkers, including multiple interleukins, interferon gamma (IFN-γ), TNF-α, and CRP (5, 55).

An evaluation of over 7,000 subjects in the PREDIMED (Prevention with Mediterranean Diet) trial, conducted over a median time of 4.8 years, demonstrated an association between weight gain and increased consumption of refined grains, red meat, potatoes, alcohol, processed meat, white bread, and sweets. Increased waist circumference was associated with increased consumption of snacks, fast-food and pre-prepared dishes, processed meat, alcohol, and sweets (56). Individuals with obesity instructed to follow an energy-restricted MedDiet in the PREDIMED-Plus cohort lost more weight on average than individuals on a standard MedDiet after 1 year (57). A cross-sectional study of 62 individuals with overweight or obesity reported an association of better cardiorespiratory fitness and adherence to a MedDiet with lower blood pressure and lower body fat composition (58). For individuals with genetic risk factors for obesity, those with higher adherence to the MedDiet were less likely to develop obesity in 7–15 years of follow-up (59). A sub-study of the PREDIMED trial examining changes in inflammatory markers after 3 years of MedDiet intervention found reduced plasma levels of several inflammatory cytokines (IL-1β, IL-6, IL-8, TNF-α, IFN-γ, hs-CRP, MCP-1, MIP-1β, RANTES, and ENA78), but these did not reflect at the gene level (60).

RCT MedDiet intervention studies consistently show lower TNF-α, IFN-γ (60–62), and fecal calprotectin (55, 63). Cannabinoids as drugs, particularly those targeting the CB_2_ receptors, have been associated with relief for a number of inflammatory disorders (64). Bourdeau-Julien et al. (65) and Forteza et al. (66) both detected increased endocannabinoids (OEA and EPEA) following MedDiet intervention in healthy volunteers of normal weight. In contrast, Tagliamonte et al. found that plasma arachidonoylethanolamide (AEA) was decreased following MedDiet intervention in individuals with overweight/obesity, which increased the oleoylethanolamide/arachidonoylethanolamide (OEA/AEA) ratio concomitantly with reduced cholesterol (54).

Olive oil, as well as other components of the MedDiet, such as fresh fruits and vegetables, contain polyphenols that have anti-inflammatory properties. In a study of multiple types of olive oil, individuals eating a diet supplemented with olive oil that contained high amounts of polyphenols had significantly improved plasma inflammatory biomarkers (decreased IL-8 and TNF-α) (67), and another study reported a connection between olive oil and reduced body weight, waist circumference, and hepatic steatosis, in subjects with metabolic syndrome. The anti-inflammatory cytokine IL-10 increased, while pro-inflammatory cytokines decreased (IL-6, IL-17, TNF-α, and IL-1β) (68).

Strengths of these randomized controlled trials include that they can control for variables that are not controlled in observational studies. For example, the PREDIMED and PREDIMED-Plus trials each compared two versions of a MedDiet: MedDiet with olive oil vs. MedDiet with nuts, or energy-restricted MedDiet vs. non-energy-restricted MedDiet (57, 60). Researchers are able to collect health information that may not be available in large observational studies, such as information on alcohol consumption, physical activity, and medication/supplement use, which can be used as exclusion criteria or taken to account in statistical analyses (55, 65, 66). Additionally, when studies provide the food for the participants, the content is known in detail and is much better controlled than when participants prepare their own food. Bourdeau-Julien et al. (65) and Forteza et al. (66) provided food to their volunteers, so they could exactly track the nutrient intake and compliance of their volunteers. Limitations of the RCTs include that most often there are a low number of participants: the studies described here had fewer than 100 study subjects, with the exception of the PREDIMED trial studies. Another limitation is that the food consumed is determined from records that are not seven days per week, so extrapolation is required to interpret the information as the individual's whole diet, and data are dependent on the accuracy and adherence of the study subjects. Most of the studies described here have a narrowly defined inclusion criteria, such as those with a specific disorder, so the results may not translate to healthy individuals or individuals with other medical conditions. Most of the studies examine the effect of the intervention over a short period of time, often weeks to several months, raising the question of whether the intervention had time to establish an effect, and whether an effect would be sustainable. However, the PREDIMED trial, which is the exception and covered a long period of time, ended after 4.8 median years of follow-up, and showed strong evidence of the benefit of the MedDiet in many areas, resulting in over 350 publications so far according to their website (69). Overall, the results of the RCTs are consistent with the results of the observational studies and the evidence supports the role of the MedDiet in modulating inflammation and obesity.

#### 3.2.3 Mechanisms of dietary effects on inflammation

Meta-analyses of multiple studies provide support for the conclusion that the MedDiet reduces the risk of obesity. For example, a meta-analysis of 15 RCTs of MedDiet interventions that measured obesity parameters in children and adolescents reported that the interventions had a significant effect on reducing BMI and obesity in this population (70). A systematic review of ten RCTs found that diets such as the MedDiet, and other similar dietary patterns, were associated with a significant reduction of CRP and an increase in adiponectin, both indicators of reduced inflammation (71). A systematic review of 20 RCTs reported the following changes in biomarkers in association with a MedDiet: decreased pro-inflammatory cytokines IL-1α, IL-1β, IL-5, IL-6, IL-7, IL-8, IL-18, IFN-γ, TNF-α, CRP, high-sensitivity CRP and increased anti-inflammatory cytokines IL-4 and IL-10 (5). A meta-analysis of 32 studies concluded that omega-3 polyunsaturated fatty acid dietary supplementation had anti-inflammatory effects, as shown by a decrease in CRP and TNF-α (72). Therefore, the anti-inflammatory effects of the MedDiet as a whole, as well as of the individual dietary components, contribute to its status as a healthy diet that may combat obesity. Data that associate the MedDiet with reduced inflammation are abundant but obtaining an understanding of the detailed pathophysiology is a more challenging goal. Some recent studies delving into the mechanisms of dietary effects on inflammation are reviewed below.

To define biological pathways affected by components of the diet, studies using murine models and *in vitro* cultures can be quite valuable (23, 73, 74), as specific mechanistic hypotheses can be generated from such studies. A study of 952 individuals using genome-wide genotyping, gut metagenomic sequence data, and fecal SCFA levels, reported that increased butyrate production was associated with impaired insulin response and that abnormal production or absorption of propionate was associated with T2D risk (75). In Section 5.1, we will discuss the evidence that SCFAs are increased in response to the MedDiet and this excellent study by Sanna et al., combined with the other literature, allow us to associate the MedDiet to SCFA changes to an impaired insulin response and obesity.

Cross-sectional studies examining adherence to the MedDiet and CRP concentrations found these to be inversely correlated (76, 77). This was also observed in a large population-based study (78). A recent study attempting to better define specific physiologic connections between obesity and inflammation used a mouse model with a CRP transgene. The investigators provided evidence that CRP is not merely a marker of inflammation, but instead has a causal role in the development of obesity (6).

As part of the CORDIOPREV (CORonary Diet Intervention with Olive oil and cardiovascular PREVention) prospective RCT, researchers suggest that the genetic variant of the NLRP3 inflammasome may modulate the benefits of the MedDiet (79). Murine and human brain cells treated with virgin olive oil reduced activation of the inflammatory TLR4/NLRP3 axis (80). Deficiency of NLRP3 attenuated systemic inflammation, especially with a HFD, caused changes in the plasma metabolome, metabolites in the liver and myocardium, and gut microbiota compared to wild-type mice (81). The saturated fats common in the Western diet are also associated with increases in inflammation by the nuclear factor kappa B (NF-κB) pathway and NLRP3 inflammasome, possibly contributing to obesity, as reviewed by Las Heras et al. (24). Even occasional consumption of Western diet patterns increased inflammation and insulin resistance in a rodent study (82). The effects of dietary patterns on health are complex and understanding their mechanisms will be necessary to use diet for the treatment of obesity and other health conditions.

## 4 MedDiet and the gut microbiome

Dietary patterns such as the Western diet and UPF consumption likely contribute to obesity partially through their impact on the gut microbiome. The gut microbiome is highly modifiable by diet and multiple studies have shown alterations to the microbiome from dietary patterns like the MedDiet (65, 83–85). Due to the complexity of the microbiome and the variation in how MedDiet is characterized between studies, it is difficult to define one consistent microbiome signature associated with the MedDiet (86). Clear changes in the microbiome have not been found during all MedDiet interventions, especially when the starting microbiome of the individual had high diversity, as the diverse microbiome was somewhat more resistant to changes (65, 87). When trying to assimilate all of the available literature on a topic, it is our view that if a meta-analysis reveals striking differences in results between various studies, this does not negate the results of each individual well-controlled study, but instead the meta-analysis demonstrates that generic conclusions about the MedDiet may not apply to every population and disease state. The discrepancies highlight our lack of understanding regarding which of the key variables in each study are most contributory to the outcome. The MedDiet can also have a considerable impact on microbial metabolites, even without a significant corresponding change to microbiome composition. Regardless of our limited understanding of the complex gut microbial communities, and their individual or overlapping roles, there are data to support beneficial changes to the microbiome from MedDiet intervention.

Several studies have investigated the role of diet in SCFA metabolism. For example, a MedDiet intervention in women with obesity was able to reverse features of dysbiosis by increasing microbiome biodiversity and SCFA-producing taxa (88). MedDiet adherence in both individuals with obesity and normal weight was positively correlated with SCFA-producing taxa such as *Bifidobacterium animalis* (89). MedDiet intervention has been reported to increase fecal SCFAs (90), and the abundance of butyrate-producing microbes (87).

Some studies have focused on changes in BAs. An 8-week RCT of 82 individuals with overweight and obesity reported that increased adherence to a MedDiet resulted in a reduction of plasma cholesterol and fecal BAs. Gut microbiome analysis revealed an increase in *Faecalibacterium prausnitzii* and decrease in *Ruminococcus gnavus*. Furthermore, there were increased urinary urolithins, fecal BAs degradation, and insulin sensitivity in subjects on the MedDiet, which correlated with specific microbial taxa (91).

Fiber is known to be a critical component of the MedDiet. Dietary fiber originates primarily from whole grains and vegetables, foods that can serve as a prebiotic for bacterial growth, but different types of fiber may have different effects. Healthy adults supplemented with resistant potato starch had increased bifidobacteria and butyrate production in their gut, while supplementation with fiber from maize and chicory root did not show a statistically significant difference. Among individuals whose microbiome changed, the highest butyrate concentrations were correlated with *Ruminococcus bromii* or *Clostridium chartatabidum* increases (92). The effect of fiber supplementation on the microbiome and SCFA production varies between individuals. The authors report that some individuals are limited in their capacity to produce SCFA from fiber supplementation, and this may be driven by their microbiome (92, 93). Another dietary intervention showed that fiber from a mixture of fruits and vegetables resulted in increased bifidobacteria but no increases in SCFAs over a short 2-week period (94).

There have been numerous studies of the MedDiet component olive oil. Mice supplemented with olive oil had microbial changes associated with reduced inflammation and the prevention of colorectal cancer compared to mice fed other fat types. Interestingly, the olive oil diet in the mice increased the Firmicutes/Bacteroidetes ratio, which correlated with lower colorectal cancer risk but higher risk of obesity in this study (95). Olive oil consumption, particularly oil enriched with phenolic compounds, was also associated with increased bifidobacteria in a RCT in individuals with high cholesterol (96). Olive oil is an important source of flavonoids, and microbial metabolism is required to make flavonoids biologically available (97).

There are a few common patterns to the changes to the microbiome that have been reported repeatedly, either in studies comparing the MedDiet to a Western diet, or in studies comparing individuals with obesity to lean controls. A study of 92 individuals found an association of overweight/obesity with specific gut microbiota patterns when compared to those of normal weight: Bacteroidetes taxa were decreased and several Firmicutes taxa were increased (98). The Western diet is associated with decreased beneficial bacteria such as bifidobacteria and eubacteria in the human gut (99) and, in rodents, decreased *Akkermansia* spp., species that are associated with a number of human diseases (100). Lean mice receiving fecal transplants from mice with obesity gain weight (101, 102) and individuals with obesity receiving transplants from lean individuals had improved metabolic disease symptoms (103). These studies show the combined value of animal and human studies. The studies make associations between the microbiome, obesity, and metabolic syndrome. The data supporting an association of the MedDiet with a reduction in inflammation and obesity from Section 3 of this review, combined with studies in Section 4 that investigated the microbiome and obesity, serve to connect the MedDiet to inflammation, obesity, and the gut microbiome. Every study does not prove direct causation, but the results allow the development of a larger hypothesis for definitive testing. Human fecal microbiome transplants have successfully altered the microbiomes of individuals with obesity to resemble lean donors, however no change in BMI occurred over the 12-week study. The time required to significantly change the BMI may be longer than the time to alter the microbial community (104). Individuals with obesity have distinct microbial communities, often characterized by having an increased ratio of Firmicutes to Bacteroides compared to lean individuals and decreased microbial diversity (98, 102) although these results are not consistent across all studies (105).

Gut permeability and energy efficiency are two other elements that have been examined closely. The microbiomes of individuals with obesity may result in increased energy absorption from food. Increases in Firmicutes relative to Bacteroides elevate levels of alpha amylases and amylomaltases for more efficient energy extraction from foods, which increases the number of calories absorbed (102). An imbalanced microbiome can also contribute to obesity through its role in inflammation. The dysbiosis of obesity can lead to increased gut permeability and allow proinflammatory molecules to enter systemic circulation. The microbiome of humans and mice with obesity reduced the expression of the zonula occludens-1 tight junction protein, weakening the gut barrier (106). Individuals with obesity also have increased Gram-negative bacterial taxa of the *Enterobacteriaceae* family in their microbiome, resulting in elevated levels of LPS which can leak from the gut (98). LPS is proinflammatory and promotes low grade inflammation which promotes the storage of excess lipids (107). Further discussion of LPS as it relates to the MedDiet is included in Section 5.4 of this review.

By combining all of the findings from the many investigations discussed above, a positive role of the MedDiet on obesity and inflammation seems quite clear. We have yet to obtain a detailed understanding of the pathophysiology of obesity, but recent work has started to dissect the role of specific gut microbial metabolites in these pathways.

## 5 Interplay of obesity, the MedDiet, and gut-derived metabolites

Obesity is associated with changes in the composition of the gut microbiota, and in the amounts and types of microbial metabolites that are formed. Two groups of metabolites of demonstrated importance in obesity physiology are SCFAs and BAs. An increase in a third gut-derived metabolite, TMAO, has been associated with obesity and inflammation; however, its effects are proposed to be context-dependent (108, 109). A fourth metabolite associated with inflammation and obesity is LPS. Obesity has been associated with increased intestinal permeability, which allows the movement of bacteria and bacterial products, like LPS, into the bloodstream with an associated increase in inflammation (110). The interactions between the obese gut microbiota, gut-derived metabolites, and the effects on its host are quite complex and multifactorial.

Previous reviews indicate that adults with obesity have been shown to have increased total concentrations of fecal SCFAs (11, 12) and BAs (13, 14), likely due to dysregulated metabolism and absorption. However, analysis of the gut microbiota of over 1,900 individuals in the METS-microbiome study showed an association of obesity with a reduction of fecal SCFA concentrations, gut microbial diversity, and of the bacteria that synthesize SCFAs, while the country of origin for the study subjects was the most important variable. Using predictive modeling, SCFA concentrations could not predict obesity status, suggesting the relationship between SCFAs and obesity is still unclear (111). Many of the studies examining SCFAs in populations with overweight/obesity have been cross-sectional analysis, with or without disease comorbidities and/or medications, and using different biospecimen types (fecal vs. blood), making it difficult to draw definitive conclusions (112–114). Meanwhile, the clinical controlled trials measuring SCFAs in populations with obesity also apply various pre/probiotic, dietary, or weight-loss interventions which make comparing studies difficult (115–117). Several variables such as diet and physical activity can affect SCFA production, and the direction of change for individual SCFAs (i.e., acetate vs. butyrate vs. propionate, etc.) likely differ, as is observed in Table 2, and should be considered when comparing data between studies.

Few studies have examined SCFA levels in children with obesity, however, within the last 5 years, two studies showed increased fecal SCFA concentrations (118, 119), while one study showed fecal SCFAs were reduced (120) in children with obesity. The differences in study results may be due to study design and inclusion criteria, as Wei et al. and Gyarmati et al. excluded volunteers who had received antibiotic, prebiotic, or probiotic treatments within the last 3 months before the studies, while the study by Slizewska et al. did not (118–120).

In Tables 1–3 we have summarized some of the recent human studies that have investigated changes in gut metabolites in association with a MedDiet compared to other diets. Below, we discuss the effects of MedDiet on SCFAs, BAs, TMAO, and LPS, and the mechanisms by which the MedDiet could potentially alter the gut microbiota to combat obesity.

**Table 1:** Characteristics of recent clinical studies investigating the effects of the MedDiet on gut-derived metabolites.

**References**	**Country**	**Study design (cohort)**	**Study population**	**MedDiet intervention**	**Control**	**Sample size (*n*)**	**Sex (*n*)**	**Age (*y*: mean ± SD, or CI, and/or range)**	**BMI (kg/m^2^: mean ± SD, or CI, and/or range)**	**Duration**	**MedDiet score**
André et al. (121)	France	Cross-sectional (Alienor Study, subsample of 3C Study)	French older community-dwelling adults	N/A	Traditional dietary pattern	698	266 M; 432 F	73.1 ± 4.4	26.3	N/A	8-Item Study specific score from FFQ
Baratta et al. (41)	Italy	Observational cohort study (PLINIO Study)	Patients with NAFLD (now MASLD)	N/A	N/A	238	135 M; 103 F	53.1 ± 12.4	31.2 ± 5.4 sNox2-dp tertile I; 30.3 ± 4.1 II; 29.4 ± 4.2 III	N/A	9-item Mediterranean-diet questionnaire (122)
Barber et al. (87)	Spain	Randomized cross-over (N/A)	Healthy men	Fiber-enriched MedDiet	Western-type diet	20	20 M; 0 F	18–38	19.2–25.5	2 Mo (2 W each diet)	Food was provided
Barrea et al., (123)	Italy	Cross-sectional (N/A)	Healthy adults	N/A	N/A	144	67 M; 77 F	31.55 ± 6.19	22.84 ± 1.51	N/A	14-point Mediterranean Diet Adherence Screener (MEDAS) from PREDIMED Study (124)
Barrea et al. (125)	Italy	Cross-sectional (OPERA Project)	Healthy Caucasian adults	N/A	N/A	247	100 M; 147 F	36.6 ± 11.0	28.8 ± 9.1; 19–59	N/A	14-point Mediterranean Diet Adherence Screener (MEDAS) from PREDIMED Study (126)
Barrea et al. (127)	Italy	Case-control, cross-sectional (OPERA Project)	Patients with Hidradenitis Suppurativa (HS) and healthy controls	N/A	N/A	70	22 M; 48 F	25.37 ± 8.36 HS; 26.14 ± 7.28 healthy	29.26 ± 5.33 HS; 29.22 ± 5.62 healthy	N/A	14-point Mediterranean Diet Adherence Screener (MEDAS) from PREDIMED Study (126)
Bourdeau-Julien et al. (65)	Canada	Fixed-sequence (N/A)	Healthy adults	MedDiet	CanDiet	21	10 M; 11 F	20–29 M; 20–34 F	20.4–25 M; 20.1–24.1 F	19 D (3 D MedDiet, then 13 D CanDiet, then 3 D MedDiet)	Food was provided
De Filippis et al. (128)	Italy	Cross-sectional (N/A)	Healthy adults	N/A	Omnivore = Western diet	153	64 M; 89 F	39 ± 9 vegetarian; 37 ± 10 vegan; 37 ± 9 omnivore	21.9 ± 2.5 vegetarian; 21.3 ± 2.2 vegan; 22.1 ± 2.0 omnivore	N/A	11-unit dietary score based on tertiles (129)
Forteza et al. (66)	Canada	Randomized Cross-over (N/A)	Healthy, physically-active women	MedDiet	CanDiet (Western-type)	7	0 M; 7 F	25 ± 5; 19–32	22.52 ± 1.57; 19.50–24.49	35 D (7 D per diet)	Food was provided
Galie et al. (130)	Spain	Randomized cross-over (METADIET)	Adults with overweight/ obesity and metabolic syndrome	MedDiet plus mixed nuts (50 g/day)	Habitual diet supple- mented with nuts (50 g/day)	44	NR	25–60	25–35	5 Mo (2 Mo each diet + 1 Mo washout)	17-point MedDiet score used in PREDIMED-Plus (131)
Garcia-Mantrana et al. (132)	Spain	Cross-sectional (N/A)	Healthy adults	N/A	N/A	27	11 M; 16 F	39.5 ± 7.3	25.29 ± 2.76 M; 21.95 ± 2.72 F	N/A	14-point Mediterranean Diet Adherence Screener (MEDAS) from PREDIMED Study (124)
Ghosh et al. (42)	UK, France, Netherlands, Italy & Poland	Randomized parallel (NU-AGE Study)	Elderly non-frail adults	MedDiet tailored for elderly (Nu-AGE diet)	Habitual diet	612	286 M; 326 F	65–79	18.5–46	12 Mo	Adherence scores to the MedDiet calculated based on the NU-AGE Food Based Dietary Guidelines (FBDG) (133)
Griffin et al. (134)	USA	Randomized parallel (Healthy Eating Study for Colon Cancer Prevention)	Healthy adults at increased risk for colon cancer	MedDiet	Healthy Eating diet	115	32 M; 83 F	52 ± 12	27.0 ± 3.7	6 Mo	7-item Self-Efficacy score (not specific to MedDiet) (135)
Guasch-Ferre et al. (136)	Spain	Randomized parallel (PREDIMED Study)	Community-dwelling adults at high risk for CVD	MedDiet + EVOO or MedDiet + mixed nuts	Control diet (reduce intake of all types of fat)	980	442 M; 538 F	67.5 ± 10.9	29.6 ± 3.6	12 Mo	Not provided
Gutierrez-Diaz et al. (137)	Spain	Cross-sectional (N/A)	Healthy adults	N/A	N/A	31	8 M; 23 F	42.1 ± 10.9	26.3 ± 4.7 MDS ≥4; 26.2 ± 5.0 MDS < 4	N/A	8 point Mediterranean diet score (138, 139)
Haskey et al. (55)	Canada	Randomized parallel (N/A)	Adults with ulcerative colitis (UC)	MedDiet	Habitual CanDiet	28	10 M; 18 F	18–65 MedDiet; 25–64 CanDiet	17–30 MedDiet; 19–29 CanDiet	3 Mo	24 Point Mediterranean Diet Serving Score (140)
Krishnan et al. (141)	USA	Randomized cross-over (N/A)	Adults with overweight/ obesity	MedDiet + 200 g red meat/week	MedDiet + 500 g red meat/week	39	12 M; 77 F	30–69	30.5 ± 0.3; 25–37	14 W (5 W per diet)	Food was provided
Maldonado-Contreras et al. (142)	USA	Cross-sectional (N/A)	Caribbean Latino older adults	N/A	N/A	20	6 M; 14 F	62.7 ± 8.1	28.9 ± 4.9	N/A	9-point MedDiet score (MDS) modified from (143)
Meslier et al. (91)	Italy	Randomized parallel (N/A)	Healthy adults with overweight/ obesity and sedentary lifestyle	MedDiet tailored to individual energy intake	Volunteers who maintained their regular diets	82	39 M; 34 F	43 ± 12	31.1 ± 4.5	2 Mo	11-item Italian Mediterranean Index (129)
Mitsou et al. (144)	Greece	Cross-sectional (N/A)	Healthy adults	N/A	Low MedDiet score (assumed Western diet)	120	61 M; 55 F	41.27 ± 13.33	27.29 ± 4.48	N/A	11-item MedDiet score (145)
Nagpal et al. (146)	USA	Randomized cross-over (N/A)	Older adults with mild cognitive impairment and cognitively normal controls	Modified Mediterranean-Ketogenic diet (MMKD)	American Heart Association Diet (AHAD)	17	5 M; 12 F	64.6 ± 6.4	NR	18 W (6 W each diet + 6 W washout)	Extra virgin olive oil was supplied to volunteers and ketones were measured weekly
Pagliai et al. (90)	Italy	Randomized cross-over (CARDIVEG Study)	Healthy adult Caucasian omnivores with overweight/ obesity and low-to-moderate cardiovascular risk	Hypocaloric MedDiet	Hypocaloric vegetarian diet	23	7 M; 16 F	58.6 ± 9.8	31.06 ± 0.67 MedDiet; 30.10 ± 0.61 vegetarian	6 Mo (3 Mo per diet)	9-item MedDiet Adherence Score in CARDIVEG Study (147)
Park et al. (148)	USA	*Post-hoc* analysis of randomized cross-over (N/A)	Healthy adults	Moderate fat MedDiet (South Beach)	High fat (Atkins), low fat (Ornish)	14	NR	30.6 ± 9.6	22.6 ± 3	20 W (4 W per diet)	N/A
Pastori et al. (149)	Italy	Prospective (N/A)	Adults with atrial fibrillation	N/A	N/A	912	521 M; 391 F	73.5 ± 8.3	27.5 ± 4.7	Median follow-up 40.0 (20.5-68.0) Mo	A 9-item MedDiet validated survey (122)
Pastori et al. (150)	Italy	*Post-hoc* analysis of a prospective study (N/A)	Adults with atrial fibrillation	N/A	N/A	907	516 M; 391 F	73.5 ± 8.2	NR	Median follow-up 40.5 Mo	A 9-item MedDiet validated survey (122)
Pignanelli et al. (151)	Canada	Cross-sectional (N/A)	Adults with atherosclerosis	Educated about MedDiet	N/A	276	164 M; 112 F	66.87 ± 10.45	28.49 ± 6.08	N/A	8-point Mediterranean (aMED) diet scores from the FFQ (143, 152)
Quercia et al. (153)	Italy	*Post-hoc* of randomized Parallel (N/A)	Adults with reactive hypoglycemia (RH) and healthy adults	MedDiet and Ma-Pi 2 diet designed for hypoglycemia	Free MedDiet consumed by healthy controls	19	NR	27–65 RH; 25–36 healthy	21.7–37.4 RH; 20–23.4 healthy	3 D	Food was provided
Ruiz-Saavedra et al. (154)	Spain	Cross-sectional (N/A)	Healthy older adults	N/A	N/A	73	20 M; 53 F	56–95	19.9–37.5	N/A	Mediterranean adapted Diet Quality Index-International (DQI-I) (155) Modified Mediterranean Diet Score (MMDS) (156) Relative Mediterranean Diet Score (rMED) (157) • All calculated from FFQ
Seethaler et al. (158)	Germany	Randomized parallel (LIBRE Study)	Women with BRCA1 and/or BRCA2 gene mutations and intestinal barrier impairment	MedDiet	Standard diet	260	0 M; 260 F	43.9 (CI: 42, 46) MedDiet; 44.8 (CI: 43, 46) control	25.0 (CI: 24, 26) MedDiet; 25.0 (CI: 24, 26) control	3 Mo	14-point Mediterranean Diet Adherence Screener (MEDAS) from PREDIMED Study (159) translated into German and re-validated (160) FFQ MedDiet score provided using the adapted Mediterranean Diet Score (MedD-Score) according to Trichopoulou et al. (143)
Seethaler et al. (161)	Germany	Randomized parallel (LIBRE Study)	Women with BRCA1 and/or BRCA2 gene mutations	MedDiet	Standard diet	68	0 M; 68 F	42 (CI: 35, 49) MedDiet; 41 (CI: 35, 50) control	23 (20, 27) MedDiet; 24 (CI: 21, 28) control	12 Mo	14-point Mediterranean Diet Adherence Screener (MEDAS) from PREDIMED Study (159) translated into German and re-validated (160) FFQ MedDiet score provided using the adapted Mediterranean Diet Score (MedD-Score) according to Trichopoulou et al. (143)
Shankar et al. (162)	USA & Egypt	Cross-sectional (N/A)	Healthy preadolescent and adolescent males	N/A	U.S. teenagers consuming Western diet	42	42 M; 0 F	13.9 ± 0.6 Egyptian; 12.9 ± 2.8 American	18.9 ± 2.5 Egyptian; 21.2 ± 3.4 American	N/A	N/A
Shoer et al. (163)	Israel	Randomized parallel (N/A)	Pre-diabetic individuals	Personalized postprandial glucose-targeting (PPT) diet	MedDiet	200	87 M; 113 F	50.92 ± 8.03 MedDiet; 50.37 ± 7.86 PPT	30.86 ± 6.01 MedDiet; 30.68 ± 5.23 PPT	6 Mo intervention + 6 Mo follow-up	N/A
Strauss et al. (63)	Canada	*Post-hoc* analysis of randomized controlled clinical trial (N/A)	Patients with ulcerative colitis (UC)	MedDiet	Habitual diet	40	21 M; 19 F	21–80	19–32	2 Mo	Modified 14-question Mediterranean Diet Adherence Screener (MEDAS) for the PREDIMED study (164)
Tanaka et al. (165)	USA	Cross-sectional (BLSA cohort)	Community-dwelling older adults, who reside primarily in the Washington DC–Baltimore area	N/A	N/A	806	391 M; 415 F	73.3 ± 7.1	NR	N/A	9-item MedDiet score (143) Mediterranean–DASH Diet Intervention for Neurodegenerative Delay (MIND) score (166)
Vitale et al. (167)	Italy	Randomized parallel (N/A)	Healthy adults with overweight/ obesity	Isoenergetic MedDiet	Western-type diet (habitual control diet)	29	14 M; 15 F	41.6 ± 12.3 MedDiet; 45.9 ± 13.0 control	28.9 ± 2.3 MedDiet; 29.3 ± 3.5 control	2 Mo	Main foods provided
Zhu et al. (168)	USA	Randomized cross-over (N/A)	Healthy young adults	MedDiet	Fast-food diet	10	NR	22.1 ± 2.33	24.39 ± 3.71	12 D (4 D each diet)	Food was provided

MedDiet, Mediterranean diet; WD, Western diet; CanDiet, Canadian diet; BMI, body mass index; FFQ, food frequency questionnaire; NAFLD, nonalcoholic fatty liver disease; MASLD, Metabolic Dysfunction-Associated Steatotic Liver Disease; MEDAS, Mediterranean Diet Adherence Screener; MMDS, Modified Mediterranean Diet Score; CVD, cardiovascular disease; UC, ulcerative colitis; MDS, Mediterranean Diet Score; rMED, relative Mediterranean Diet Score; PPT, Personalized Postprandial Glucose-Targeting; FBDG, Food Based Dietary Guidelines; DQI-I, Diet Quality Index-International; PPGR, Postprandial Glycemic Response; MACE, Major Adverse Cardiovascular Event; DASH, Dietary Approaches to Stop Hypertension; MIND, Mediterranean-DASH Intervention for Neurodegenerative Delay; HS, Hidradenitis Suppurativa; EVOO, extra virgin olive oil; RH, reactive hypoglycemia; HS, Hidradenitis Suppurativa; D, days; W, weeks; Mo, months; M, male; F, female; CI, 95% confidence interval; MMKD, Mediterranean-Ketogenic diet.

**Table 2:** Results summary of metabolite changes in recent clinical studies investigating associations between gut-derived metabolites and the MedDiet.

**References**	**Specimen type**	**Direction of change in metabolites relative to Med-like diet adherence**				
		**SCFA/BCFA**	**BA**	**TMAO**	**LPS**	**Other metabolites**
André et al. (121)	Blood (plasma)	—	—	—	↓ MedDiet (*p* = 0.03); ↓ Prudent diet (*p* = 0.01); ↑ Traditional diet (*p* = 0.04); ↔ Complex Carbohydrate diet (*p* = 0.41)	—
Baratta et al. (41)	Blood (serum)	—	—	—	↑ with ↓ MedDiet by association; ↑ LPS = ↑ sNox2-dp (tertile III, *p* = 0.002)	↑ sNox2-dp (tertile III) = ↓ wine (*p* = 0.046) and ↓ fish (*p* = 0.030) according to MedDiet score
Barber et al. (87)	Urine	—	—	↑ (1.5-fold) after MedDiet	—	↑ deoxycholate glucuronide (2.1-fold), 5-hydroxyindole (2-fold), L-aspartyl-L-phenylalanine (2.4-fold) after MedDiet
Barrea et al., (123)	Blood (serum)	—	—	↓ (*p* < 0.001 M; *p* = 0.002 F) with MedDiet adherence	—	—
Barrea et al. (125)	Blood (plasma)	—	—	↓ (*p* < 0.001) with MedDiet adherence	—	—
Barrea et al. (127)	Blood (serum)	—	—	↓ by association with MedDiet adherence	—	—
Bourdeau-Julien et al. (65)	Blood (serum)	↑ valerate after CanDiet vs. first MedDiet (*p* < 0.01); ↓ valerate after second MedDiet vs. CanDiet (*p* < 0.05); ↑ BCFAs isobutyrate & isovalerate after CanDiet vs. first MedDiet (*p* < 0.05); ↓ BCFAs isobutyrate & isovalerate after second MedDiet vs. CanDiet (both *p* < 0.05)	—	—	—	↑ ECs after first MedDiet vs. baseline (DHEA, *p* < 0.01; EPEA, *p* < 0.05; 2-DHG, *p* < 0.01; 2-EPG, *p* < 0.01); ↓ ECs after CanDiet vs. first MedDiet (DHEA, *p* < 0.001; EPEA, *p* < 0.01; OEA, *p* < 0.05; 2-DHG, *p* < 0.001; 2-EPG, *p* < 0.001; 2-OG, p < 0.01); ↑ ECs after second MedDiet vs. CanDiet (DHEA, *p* < 0.001; EPEA, *p* < 0.05; OEA, *p* < 0.01; 2-DHG, *p* < 0.001; 2-EPG, *p* < 0.01; 2-OG, p < 0.05)
De Filippis et al. (128)	Feces (SCFA), urine (TMAO)	↑ butyrate, propionate, acetate (*p* < 0.01), and ↓ valerate (*p* < 0.05), with high MedDiet adherence vs. low MedDiet adherence	—	↓ TMAO in vegetarian and vegan diets compared to omnivores	—	Several significant metabolites in Table S3 of original article
Forteza et al. (66)	Blood (plasma)	↑ acetic acid and ↓ isovaleric acid after MedDiet before aerobic exercise (*p* < 0.05)	—	—	—	↑ EC OEA after MedDiet before and during exercise; ↑ ECs AEA (*p* < 0.05) and EPEA (*p* < 0.001) after MedDiet immediately after exercise
Galie et al. (130)	Blood (plasma)	—	TLCA and GUDCA positively associated with MedDiet; TCA negatively associated with MedDiet	TMA positively associated with MedDiet	—	See Table 2 in original article for all 65 metabolite results
Garcia-Mantrana et al. (132)	Feces	↑ acetate + propionate + butyrate (*p* = 0.023) with MedDiet; ↑ acetate (*p* = 0.006; *p* = 0.001), propionate (*p* = 0.016; *p* = 0.004), and total SCFA (*p* = 0.020; *p* = 0.003) with vegetal proteins and polysaccharides, respectively	—	—	—	—
Ghosh et al. (42)	Blood (plasma)	↑ SCFAs & BCFAs inferred with positive microbiome changes	↑ CA (*p* < 0.006), GCDCA (*p* < 0.006) and ↓ CDCA (*p* < 0.03) with MedDiet OTUs	—	—	—
Griffin et al. (134)	Blood (serum)	—	—	↔ TMAO, choline, carnitine, betaine, γ-butyrobetaine after MedDiet and healthy eating	LPB positively associated with TMAO	—
Guasch-Ferre et al. (136)	Blood (plasma)	—	—	↑ after MedDiet + EVOO; ↔ after MedDiet + Nuts	—	—
Gutierrez-Diaz et al. (137)	Feces	↑ butyrate (*p* = 0.018) & propionate (*p* = 0.034), in MDS ≥ 4 vs. MDS < 4	—	—	—	—
Haskey et al. (55)	Feces	↑ total SCFAs (*p* = 0.01), acetic acid (*p* = 0.03), butryric acid (*p* = 0.03), and valeric acid (*p* = 0.008) after MedDiet vs. CanDiet	—	—	—	↓ FCP after MedDiet vs. CanDiet (*p* = 0.01); ↑ fecal sIgA after MedDiet vs. baseline (*p* = 0.004)
Krishnan et al. (141)	Blood (serum)	—	—	↑ after MedDiet + 500 g red meat vs. MedDiet + 200 g red meat (*p* < 0.001), but ↔ choline, betatine, and carnitine	—	—
Maldonado-Contreras et al. (142)	Feces	↓ acetate (*p* = 0.08) and butyrate (*p* = 0.08) with ↑ MedDiet score	—	—	—	—
Meslier et al. (91)	Feces (SCFA, BAs), blood/plasma (TMAO, carnitine, choline, creatinine, betaine), urine (TMAO, carnitine, choline, creatinine, betaine)	↔ acetate, butyrate, and propionate after MedDiet; ↓ BCFAs at 4 weeks (valerate, *p* = 0.04; 2-methylbutyrate, *p* = 0.003) or 8 weeks (isovalerate, *p* = 0.004; isobutyrate, *p* = 0.007) after MedDiet	↓ total BAs (*p* = 0.0001), total 1st BAs (*p* = 0.04), total 2nd BAs (*p* = 0.0009) DCA, and LCA after 8 weeks of MedDiet	↓ carnitine after MedDiet (*p* < 0.001)	—	↑ Total urolithins (*p* = 0.033) and urolithin-A-glucuronide (*p* = 0.025) after MedDiet
Mitsou et al. (144)	Feces	↑ acetate (*p* = 0.009) and ↓ valerate (*p* = 0.014) with high MedDiet adherence	—	—	—	—
Nagpal et al. (146)	Feces	↑ butyrate (*p* < 0.05) after MMKD	—	—	—	—
Pagliai et al. (90)	Feces	↑ propionic acid (*p* = 0.034) in MedDiet vs. vegetarian diet	—	—	—	Propionate negatively correlated with IP-10, IL-12 (*p* < 0.05), and VEGF (*p* < 0.01); acetic acid negatively correlated with IP-10, IL-10, IL-17 (p < 0.05), VEGF, and IL-12 (*p* < 0.01); butyric acid negatively correlated with VEGF, MCP-1 (*p* < 0.05), IL-12 and IL-17 (*p* < 0.01); isovalerate with IL-1RA (*p* < 0.05); isobutyric acid with IL-1RA and MCP-1 (*p* < 0.05) after MedDiet
Park et al. (148)	Plasma	—	—	↔ after Med-like vs. baseline or high fat diet	—	↓ AA valine (*p* ≤ 0.05) in Med-like diet compared to high fat diet; ↑ valine (*p* = 0.004) and leucine (p = 0.01) with high fat diet vs. baseline
Pastori et al. (149)	Blood (serum)	—	—	—	MedDiet score predictor for log-LPS (p < 0.001); ↓ LPS with ↑ fruit (*p* = 0.009), ↑ legumes (*p* = 0.005) and ↓ trend meat (0.085)	↑ TxB2 with ↑ MACE (*p* < 0.001); log-LPS (*p* < 0.001) and MedDiet score (*p* < 0.001) associated with TxB2
Pastori et al. (150)	Blood (plasma)	—	—	—	↑ with ↓ MedDiet adherence by association	↑ PCSK9 with ↓ MedDiet adherence (*p* = 0.001), especially ↓ EVOO (*p* = 0.001) and ↓ moderate wine consumption (*p* = 0.007)
Pignanelli et al. (151)	Plasma	—	—	—	—	p-cresyl sulfate, hippuric acid, indoxyl sulfate, p-cresyl glucuronidate, phenyl acetyl glutamine, and phenyl sulfate did not correlate with MedDiet
Quercia et al. (153)	Feces	↔ butyrate (*p* = 0.2), propionate (*p* = 0.5), or acetate (*p* = 0.5) with MedDiet vs. baseline; ↑ butyrate, propionate, and acetate with vegan diet vs. baseline (all *p* < 0.01)	—	—	—	—
Ruiz-Saavedra et al. (154)	Feces	butyric acid (*p* < 0.012), propionic acid (*p* = 0.001), and acetic acid (*p* < 0.001) positively associated with MMDS	—	—	—	↑ IL-8 with ↑ scores on MedDiet indices (rMed, *p* = 0.018; MMDS, *p* = 0.017)
Seethaler et al. (158)	Feces (SCFAs), blood/plasma (LBP)	↑ propionate (*q* < 0.001, +19%), ↑ butyrate (*q* < 0.001, +44%) after MedDiet vs. baseline; ↑ propionate (*p* = 0.09) after MedDiet vs. control	—	—	↓ LBP (*q* < 0.001, −6%) after MedDiet vs. baseline	↓ zonulin (*q* < 0.001, −30%) after MedDiet vs. baseline
Seethaler et al. (161)	Feces (SCFAs), blood/plasma (LBP)	NR	—	—	↓ LBP (*p* < 0.001) after MedDiet vs. baseline; ↓ LBP (*p* = 0.017) after MedDiet vs. control	↓ zonulin (*p* < 0.01) after MedDiet vs. baseline
Shankar et al. (162)	Feces	↑ propionate (*p* < 0.05) in Egyptian vs. American	↓ BAs (*p* < 0.05) in Egyptian vs. American	↓ choline (*p* < 0.01) in Egyptian vs. American	—	↑ nucleotides [hypoxanthine (*p* < 0.01) and uracil (*p* < 0.05)] in Egyptian; ↓ amino acids [aspartate, isoleucine, leucine, lysine, tyrosine, valine (all *p* < 0.01)] in Egyptian
Shoer et al. (163)	Blood (serum)	↑ butyrate-related compounds after PPT diet	—	—	—	↑ 10 uncharacterized biochemicals, 7 lipids, 6 AA, 1 xenobiotic (3-bromo-5-chloro-2,6- dihydroxybenzoic acid), 1 peptide (HWESASXX), 1 nucleotide (dihydroorotate) and bilirubin after MedDiet
Strauss et al. (63)	Feces	↑ valerate (*p* = 0.05), ↔ acetate, propionate, and butyrate after MedDiet vs. habitual diet	↑ GCDCA (*p* = 0.02), ↔ CA, CDCA, and DCA after MedDiet vs. habitual diet	—	—	↓ FCP associated with ↑ MedDiet score (*p* = 0.004)
Tanaka et al. (165)	Blood (plasma)	—	DCA (MDS, *p* = 0.04; MIND, *p* = 0.004); GUDCA (MDS, *p* = 0.04; MIND, *p* = 0.001); GCDCA (MIND, *p* = 0.05); GDCA (MIND, *p* = 0.04) with MedDiet indices Other NS bile acids reported in Table S4 within the original article.	Not correlated with MDS	—	↑ or ↓ TG with ↑ MedDiet adherence
Vitale et al. (167)	Blood (serum)	↑ butyric acid IAUC in MedDiet group (*p* = 0.019)	—	—	—	↓ LDL-cholesterol in MedDiet group (*p* = 0.04)
Zhu et al. (168)	Blood (plasma)	—	No significant changes	No significant changes	—	kynurenine to tryptophan ratio ↓ after FF diet and ↑ after MedDiet (*p* = 0.005); ↑ indole-3-lactic acid (*p* = 0.003) and indole-3-propionic acid after MedDiet

↑, increase; ↓, decrease; ↔, no change; SCFA, Short Chain Fatty Acid; BA, Bile Acid; BCFA, Branch Chain Fatty Acid; TMAO, Trimethylamine N-oxide; LPS, lipopolysaccharide; FA, Fatty Acid; MedDiet, Mediterranean Diet; CanDiet, Canadian Diet; EC, Endocannabinoid; DHEA, N-docosahexaenoyl-ethanolamine; EPEA, N-eicosapentaenoyl-ethanolamine; EPG, 1/2-eicosapenaenoylglycerol; OEA, N-oleoyl-ethanolamine; DHG, 1/2-docosahexaenoyl-glycerol; OG, 1/2-oleoyl-glycerol; AEA, anandamide; CA, cholic acid; GCDCA, glycochenodeoxycholic acid; CDCA, chenodeoxycholic acid; LPB, Lipopolysaccharide Binding Protein; EVOO, Extra Virgin Olive Oil; LDL, Low-Density Lipoprotein; PPT, Personalized Postprandial Glucose-Targeting; MCP-1, Monocyte Chemoattractant Protein-1; PCSK9, Proprotein Convertase Subtilisin/Kexin type 9; FCP, Fecal Calprotectin; DCA, Deoxycholic Acid; MDS, Mediterranean Diet Score; MIND, Mediterranean-DASH Intervention for Neurodegenerative Delay; GUDCA, Glycoursodeoxycholic Acid; GDCA, Glycodeoxycholic Acid; TLCA, Taurolithocholic acid; TCA, Taurocholic acid; TG, Triglyceride; IAUC, Incremental Area Under the Curve; FF, Fast Food; MMDS, Modified Mediterranean Diet Score; rMED, relative Mediterranean Diet Score; MACE, major adverse cardiovascular event; TxB2, urinary 11-dehydro-thromboxane B2; AA, amino acids; MMKD, Mediterranean Ketogenic diet; OTU, operational taxonomic unit.

**Table 3:** Results summary of microbiome and other changes in recent clinical studies investigating associations between gut-derived metabolites and the MedDiet.

**Reference**	**Microbiome composition**	**Other health-related changes**	**Main results**
André et al. (121)	—	—	Greater adherence to Mediterranean and prudent diets associated with lower circulating 3-OH FAs.
Baratta et al. (41)	—	↑ sNox2-dp in NAFLD (MASLD); ↑ sNox2-dp = ↑ GGT, AST, ALT (all *p* < 0.001);	In NAFLD (MASLD) patients, highest sNox2-dp tertile associated with highest LPS tertile and low adherence to MedDiet (esp. wine and fish).
Barber et al. (87)	↑*Agathobaculum* spp., *Anaerostipes* spp., *Anaerostipes hadrus, Agathobaculum butyriciproducens* with MedDiet	↑ flatulence (*p* = 0.048), borborigmi (*p* = 0.016), stool consistency (*p* = 0.014), stool weight (*p* < 0.001), colonic content (*p* < 0.001) after MedDiet	MedDiet, associated with higher gas and larger colonic content, changed microbial metabolism, but less dramatically in volunteers with higher beta-diversity.
Barrea et al. (123)	—	TMAO positively correlated to BMI, WC, total cholesterol, LDL cholesterol, TG (each *p* < 0.001)	Women, who consumed more plant protein and ω-3 PUFA, had higher adherence to MedDiet and lower TMAO levels than men.
Barrea et al. (125)	—	↑ TMAO with ↑ BMI and ↓ physical activity (each *p* < 0.001); ↑ TMAO with evening chronotype (*p* < 0.001)	Morning chronotype had significantly lower BMI, WC, TMAO levels, and highest adherence to MedDiet.
Barrea et al. (127)	—	↑ TMAO in HS (*p* < 0.001) and ↓ MedDiet score in HS (*p* = 0.002)	HS patients, esp. with highest disease severity, had increased inflammation, TMAO levels, and lower adherence to MedDiet compared to healthy controls.
Bourdeau-Julien et al. (65)	↑*Bacteroides* spp., *Butyricoccus* spp., *Coprococcus.1* spp., *Lachnoclostridium* spp., *Lachnospiraceae UCG 001* spp., *Parasutterella* spp., and *Lachnospira* spp. with MedDiet	—	Lead-in MedDiet and CanDiet both showed immediate and reversable metabolite (SCFA, BCFA, EC) changes, which correlated with changes in gut microbiota composition. BCFAs more strongly reduced after second MedDiet. Higher initial gut microbiota diversity resulted in more stable microbiota response.
De Filippis et al. (128)	↑*Prevotellaceae* with plant-based diets; ↑ Bacteroidetes in vegans and vegetarians compared with omnivores (*p* < 0.05); ↑ F/B ratio in omnivores	—	Consumption of plant-based diets, associated with high MedDiet adherence, increased levels of SCFA and altered gut microbiota composition.
Forteza et al. (66)	↑*Oscillospiraceae* (*p* = 0.039) and *Prevotellaceae* (*p* = 0.047) after MedDiet vs. CanDiet	—	Consumption of short-term MedDiet vs. CanDiet leads to differential response in EC and SCFA metabolites before or immediately following acute maximal aerobic exercise.
Galie et al. (130)	Cluster of *Lachnospiraceae* spp., *Ruminococcaceae UCG002* spp., *Lachnoclostridium* spp., and *Prevotellaceae* positively associated with changes in metabolites C16-OH, C12:0, C12-OH, PC35:1, PC40:6, TGs 56:6, 46:7, 56:5, and ChoE 20:5, while negatively associated with changes in phosphoethanolamine and taurine	↓ glucose (*p* = 0.02), insulin (*p* = 0.01), and HOMA-IR (*p* = 0.01) after MedDiet	MedDiet, rather than consumption of nuts in context with a non-MedDiet, was associated with a plasma metabolic profile related to metabolic disease improvements.
Garcia-Mantrana et al. (132)	↑*Catenibacterium* spp. with high MedDiet adherence; ↑*Butyricimonas* spp., *Desulfovibrio* spp., and *Oscillospira* spp. with BMI < 25; ↓ trend F/B ratio (*p* = 0.057) with higher MedDiet score	—	Dietary habits, adherence to MedDiet pattern, and BMI affect gut microbiome and metabolite changes in healthy adults. MedDiet adherence associated with increased SCFA, *Catenibacterium* spp., and higher intake of vegetable proteins and polysaccharides.
Ghosh et al. (42)	↑*Faecalibacterium prausnitzii, Roseburia* spp. (*R. hominis* and some unclassified), *Eubacterium* spp. *(E. rectale, E. eligens, E. xylanophilum), Bacteroides thetaiotaomicron, Prevotella copri* and *Anaerostipes hadrus* with high MedDiet adherence	↓ Frailty with MedDiet (*p* < 0.06); ↓ frailty with ↑ DietPositive taxa (*p* < 0.05); ↓ hsCRP and IL-17 with DietPositive taxa	Adherence to MedDiet resulted in a changed gut microbiota and metabolites, reduced frailty, improved cognitive function, and negatively correlated with markers of inflammation.
Griffin et al. (134)	↑*Akkermansia mucinophilia* in colon biopsies with ↓ TMAO, choline, and betaine	—	No significant changes in TMAO or TMAO precursor ratios in MedDiet or Healthy Eating diet groups. Relative abundance of *Akkermansia mucinophilia* in colon biopsies negatively correlated with TMAO and some precursors (betaine, choline, carnitine).
Guasch-Ferre et al. (136)	—	↑ choline (*p* < 0.001) in cases vs. controls; baseline B/C ratio inversely associated with CVD; baseline choline metabolite score associated with a 2.21-fold higher risk of CVD across extreme quartiles (*p* < 0.001 for trend) and a 2.27-fold higher risk of stroke (*p* < 0.001 for trend)	Baseline B/C ratio negatively associated with CVD while baseline choline associated with increased risk of CVD and stroke. MedDiet associated with lower risk of CVD compared to control diet. No significant correlations between metabolites and CVD found after 1-year MedDiet intervention.
Gutierrez-Diaz et al. (137)	↑ Bacteroidetes (*p* = 0.001), *Prevotellaceae* (*p* = 0.002), and *Prevotella* spp. (*p* = 0.003); ↓ Firmicutes (*p* = 0.003) and *Lachnospiracea* (*p* = 0.045) with MDS ≥ 4 vs. MDS < 4	—	High MedDiet score associated with higher abundance of Bacteroidetes and *Prevotellacea*, and increased fecal SCFAs, propionate and butyrate.
Haskey et al. (55)	↑*Alistipes finegoldii, Flavonifractor plautii, Ruminococcus bromii* after MedDiet	—	MedDiet lowered FCP and increased SCFAs compared to CanDiet. MedDiet associated with gut microbiota species known to be protective against colitis (*Alistipes finegoldii* and *Flavonifractor plautii*) and promote the production of SCFAs (*Ruminococcus bromii*).
Krishnan et al. (141)	—	TMAO positively associated with HOMA-IR, a surrogate for insulin resistance (*p* = 0.036)	TMAO levels reduced when lower amounts of red meat (200 vs. 500 g) consumed with MedDiet.
Maldonado-Contreras et al. (142)	↑ trend *Prevotella copri* in individuals with higher 18:3 alpha linolenic fatty acid intake (*p* = 0.09); ↑ Enterobacteriales in T2D (*p* = 0.01)	butyrate (*p* = 0.03), propionate (*p* = 0.02), acetate (*p* = 0.04) correlated with % calories from fat	Caribbean Latino adults showed poor adherence to MDS or HEI-2015. Microbiome samples clustered into two groups depending on *Prevotella copri* abundance, which was related to higher alpha linolenic fatty acid intake. Individuals with T2D had higher Enterobacteriales and trend lower SCFAs.
Meslier et al. (91)	↑*Faecalibacterium prausnitzii, Roseburia* spp., and *Lachnospiraceae* after MedDiet	↓ total cholesterol 4 weeks after MedDiet (*p* < 0.05)	MedDiet increased fiber and reduced animal protein intake, reduced levels of carnitine, cholesterol, and BAs. Shotgut metagenomics showed MedDiet increased abundance of fiber-degrading *Faecalibacterium prausnitzii* and genes linked to butyrate metabolism.
Mitsou et al. (144)	↓*Escherichia coli* (*p* = 0.022), ↑ bifidobacteria:*E. coli* ratio (*p* = 0.025), and ↑*Candida albicans* (*p* = 0.039) with high MedDiet adherence	↑ total number of evacuations (*p* = 0.028), GI pain (*p* = 0.029), and bloating (*p* = 0.028) with high MedDiet adherence	High MedDiet adherence associated with lower *Escherichia coli* counts, an increased bifidobacteria: *E. coli* ratio, increased levels of *Candida albicans*, higher molar ratio of acetate, and more pronounced GI symptoms.
Nagpal et al. (146)	↓*Bifidobacteriaceae* and *Bifidobacterium* spp. after MMKD; ↑*Akkermansia* spp., Verrucomicrobia, and *Verrumicrobiaceae* after MMKD	In adults with mild cognitive impairment eating the MMKD, ↑ Tenericutes and *Enterobacteriaceae* = ↓ CSF Aβ42, ↑*Lachnospiraceae, Rikenellaeae*, and *Parabacteroides* = ↑ CSF Aβ42, and ↑*Sutterella* and *Mollicutes* = ↑ and ↓ tau-p181, respectively	MMKD can modulate the gut microbiome and serum metabolites in those at risk for Alzheimer's disease. These changes are associated with improved Alzheimer's disease biomarkers in cerebrospinal fluid.
Pagliai et al. (90)	↑*Enterorhabdus* spp. (*p* = 0.002), *Lachnoclostridium* spp. (*p* = 0.039), and ↓*Parabacteroides* spp. (*p* = 0.037) pre- vs. post-MedDiet; ↑*Clostridium sensu stricto* (*p* = 0.005), *Enterorhabdus* spp. (*p* = 0.003), *Veillonella* spp. (*p* = 0.029), and ↓*Anaerostipes* spp. (*p* = 0.048) after MedDiet vs. vegetarian diet	Anaerostipes positively correlated with LDL cholesterol and total cholesterol; HDL-cholesterol and IFN-γ^*^ negatively correlated with *Enterorhabdus* spp.; *Parabacteroidetes* spp. positively correlated with MCP-1; *Lachnoclostridium* spp. related to negative variations of IL-6, AST^*^, ALT and vitamin B12 (*p* < 0.05 or ^*^p < 0.01)	MedDiet and vegetarian diet changed some gut microbiota composition and SCFA propionate differentially. After MedDiet, variations of SCFAs negatively associated with some inflammatory cytokines (VEGF, MCP-1, IL-17, IP-10, and IL-12).
Park et al. (148)	N/A	↑ TMAO with high fat diet vs. low fat diet (*p* = 0.01)	Baseline diet and 4 -week low-fat diet reduced TMAO and BCAA levels compared to high-fat. Few changes in moderate fat Med-like diet.
Pastori et al. (149)	—	↑ LPS with ↑ MACE (*p* = 0.021), ↓ survival free of MACE (*p* = 0.001, 3rd vs. 1st LPS tertile); Log-LPS is a predictor of MACE (*p* = 0.009)	Log-LPS, age, and previous CV or cardiac events were predictors of MACE. MedDiet score (esp. higher intake of fruits and legumes) significantly affects circulating log-LPS.
Pastori et al. (150)	—	↑ of LPS (*p* < 0.001) and ↑ sNox2-dp (*p* < 0.001) with PCSK9 above the median, and these were directly correlated	LPS and PCSK9 levels significantly correlated. LPS, sNox2-dp, and high adherence to MedDiet associated with PCSK9 above the median range. Olive oil and wine intake negatively correlated with PCSK9. Patients with high levels of LPS and PCSK9 had increased incidence of CV events.
Pignanelli et al. (151)	—	↑ TMAO associated with ↓ eGFR (*p* = 0.02) or ↑ renal impairment	Impaired renal function associated with higher plasma metabolites and higher carotid plaque burden. No correlations detected between plasma metabolites and MedDiet score.
Quercia et al. (153)	Not significant	—	Gut microbiome profiles did not differ between 3-day vegan (Ma-Pi 2) and MedDiet group. SCFA levels increased only with vegan diet.
Ruiz-Saavedra et al. (154)	↑*Faecalibacterium prausnitzii* levels positively associated with DII (*p* = 0.030), HEI (*p* = 0.035), DQI-I (*p* = 0.047), and MMDS (*p* = 0.044), while *Lactobacillus* spp. levels negatively correlated with AHEI (*p* = 0.027) and MMDS (*p* = 0.012)	—	DII, HEI, DQI-I, and MMDS were positive predictors of *Faecalibacterium prausnitzii*. AHEI and MMDS were negatively associated with *Lactobacillus* spp. HEI, AHEI, and MMDS positively associated with SCFA. Lower IL-8 detected with higher MedDiet scores.
Seethaler et al. (158)	—	↓ WC (*p* = 0.005), ↓ WHR (*p* = 0.07) after MedDiet vs. control	High MedDiet adherence led to decreased LPB and zonulin levels and increased SCFAs. Propionate and butyrate identified as mechanistic links between diet and intestinal barrier integrity.
Seethaler et al. (161)	—	↑ n-3 PUFA, n-3 DHA (*p* < 0.001) with MedDiet adherence	MedDiet adherence associated with increased n-3 DHA levels and decreased LBP and zonulin levels, however the effect of n-3 DHA on intestinal barrier integrity was mild compared to SCFAs reported previously.
Shankar et al. (162)	↑*Prevotella* spp. (*p* < 0.01), *Gammaproteobacteria, Methanobacteria, Megasphaera* spp. (*p* < 0.05), *Eubacterium* spp. (*p* < 0.01), *Mitsuokella* spp. (*p* < 0.01), *Catenibacterium* spp. (*p* < 0.01), and *Succinivibrio* spp. (*p* = 0.028) in Egyptian; Egyptian = *Prevotella* spp. enterotype; American = *Bacteroides* spp. enterotype	—	The Egyptian (MedDiet pattern) gut had higher levels of SCFAs, increased prevalence/proportions of microbial polysaccharide degradation-encoding genes/genera and belonged to *Prevotella* spp. enterotype compared to American gut.
Shoer et al. (163)	↑ microbiome diversity after MedDiet (*p* < 0.05) and PPT diet (*p* < 0.01); ↑*Ruminococcaceae, Clostridiaceae, Clostridium* spp. *CAG 122* (SGB_4659, *p* = 0.01), *Clostridium* spp. (SGB_4714, *p* = 0.01), *Faecalibacterium prausnitzii* (SGB_15332, *p* = 0.03; SGB_15333, *p* = 0.008), and ↓*Eubacterium ventriosum* after MedDiet	↑ cytokines [Axin 1 (AXIN1) and Sirtuin 2 (SIRT2)] after MedDiet	PPT diet had larger impact on microbiome and metabolites (several linked to butyrate metabolism) compared to MedDiet. Oral microbiome found to be genetically more dynamic than the gut.
Strauss et al. (63)	↑*Roseburia* spp., *Lachnospiraceae* spp. and *Bifidobacterium* spp. with ↓ FCP; ↑*Bacteroides fragilis, Ruminococcus* spp., and *Eikenella corrodens* with ↑ FCP levels; ↑*Faecalibacterium prausnitzii* (*p* = 0.02), *Dorea longicatena* (NS), and *Roseburia inulinivorans* (*p* = 0.002) mediators between ↑ MedDiet and ↓ FCP	↑ benzyl alcohol, 3-hydroxyphenylacetate, 3-4-hydroxyphenylacetate and phenylacetate as mediators between ↑ MedDiet and ↓ FCP	MedDiet intervention significantly increased some SCFA and BA compared to habitual diet. Identified three taxa and four metabolites as strong mediators between MedDiet and fecal calprotectin.
Tanaka et al. (165)	—	—	All dietary scores (MDS, MIND, and AHEI-2010) inversely correlated with frailty index. Of 466 metabolites measured, 236, 218, and 278 associated with MDS, MIND, and AHEI-2010, respectively; 176 metabolites overlapped between the three diet scores. Some signatures of MIND and AHEI-2010 identified as potential mediators of diet and frailty index.
Vitale et al. (167)	↑*Intestinimonas butyriciproducens* and *Akkermansia muciniphila*; ↓*Ruminococcus torques, Coprococcus comes, Streptococcus gallolyticus* and *Flavonifractor plautii* (all *p* < 0.05) after MedDiet	—	MedDiet reduced glucose and insulin response after a meal, increased postprandial butyric acid (which negatively correlated with insulin sensitivity) and increased relative abundance of *Intestinimonas butyriciproducens* and *Akkermansia muciniphila*.
Zhu et al. (168)	↑*Butyricicoccus* spp. (*p* = 0.0001), *Lachnospiraceae_UCG-004* spp. (*p* = 0.01) after MedDiet; *Collinsella* spp. (*p* = 0.004), *Parabacteroides* spp. (*p* = 0.004), *Escherichia* spp./*Shigella* spp. (*p* = 0.03), *Bilophila* spp. (*p* = 0.03) after FF diet	—	Four-day MedDiet increased fiber-fermenting bacteria, while fast-food diet increased bile-tolerant species. Indole derivatives significantly higher after MedDiet. Interindividual variability may be due to differences in habitual diet.

↑, increase; ↓, decrease; SCFA, Short Chain Fatty Acid; BA, Bile Acid; BCFA, Branch Chain Fatty Acid; FA, Fatty Acid; TMAO, Trimethylamine N-oxide; LPS, lipopolysaccharide; BMI, Body Mass Index; NAFLD, Nonalcoholic Fatty Liver Disease; MASLD, Metabolic Dysfunction-Associated Steatotic Liver Disease; MedDiet, Mediterranean Diet; CanDiet, Canadian Diet; GGT, gamma glutamiltranspeptidase; AST, aspartate transaminase; ALT, alanine transaminase; PUFA, Polyunsaturated Fatty Acid; HS, Hidradenitis Suppurativa; EC, Endocannabinoid; F/B, Firmicutes/Bacteroidetes; LPB, Lipopolysaccharide Binding Protein; CVD, Cardiovascular Disease; HOMA-IR, Homeostasis Model Assessment-Estimated Insulin Resistance; GI, Gastrointestinal; VEGF, Vascular Endothelial Growth Factor; LDL, Low-Density Lipoprotein; HDL, High-Density Lipoprotein; PPT, Personalized Postprandial Glucose-Targeting; MCP-1, Monocyte Chemoattractant Protein-1; PCSK9, Proprotein Convertase Subtilisin/Kexin type 9; WHR, Waist-to-Hip ratio; WC, Waist Circumference; DHA, Docosahexaenoic Acid; FCP, Fecal Calprotectin; MDS, Mediterranean Diet Score; MIND, Mediterranean-DASH Intervention for Neurodegenerative Delay; AHEI, Alternative Healthy Eating Index; TG, Triglyceride; HEI, Healthy Eating Index; T2D, Type 2 Diabetes; BCAA, Branched-Chain Amino Acid; MMDS, Modified Mediterranean Diet Score; DII, Dietary Inflammatory index; DQI-I, Diet Quality Index-International; B/C, betaine/choline ratio; 1st, primary; 2nd, secondary; MACE, major adverse cardiovascular event; eGFR, estimated glomerular filtration rate; Spp., species; MMKD, Mediterranean Ketogenic diet; FF, fast food; IFN, interferon; ChoE, cholesterol ester; PC, phosphatidylcholine; CSF, cerebrospinal fluid; Aβ, amyloid beta. *Indicates *p* < 0.01.

### 5.1 Short-chain fatty acids

**Figure 1:**
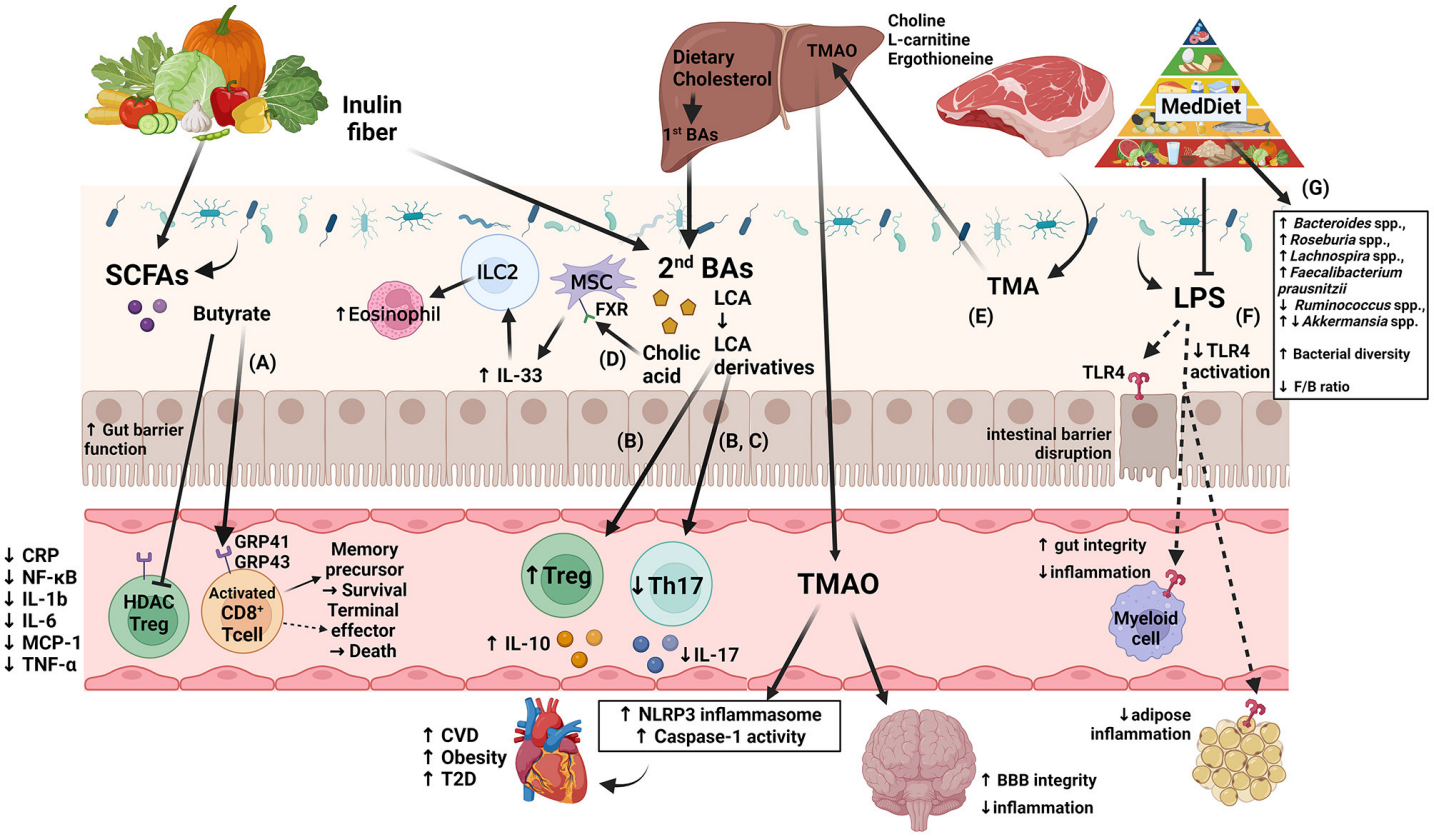
Diagram of several of the proposed immune modulation pathways that incorporate the four gut microbiota-derived metabolites: SCFA, BA, TMAO, and LPS, highlighting new findings from recent human and murine studies. **(A)** Production of SCFAs improves barrier function, Treg cell activation, and anti-inflammatory effects, as reviewed in (24, 170). A murine study showed that by uncoupling the tricarboxylic acid cycle from glycolysis, butyrate promotes the uptake and oxidation of fatty acids, leading to enhanced cellular metabolism and memory potential of activated CD8+ T-cells (171). **(B)** Another murine study showed that derivatives of secondary bile acid LCA, 3-oxoLCA and isoalloLCA, were identified as T-cell regulators. While 3-oxoLCA inhibited Th17 cell differentiation through binding to its main transcription factor, RORγt, isoalloLCA upregulates Treg cell differentiation through the production of mitoROS, which increases FoxP3 expression (172). **(C)** Using murine models and *in vitro* studies with bacteria derived from humans, several bacterial genera were identified as producers of 3-oxoLCA, including *Gordonibacter pamelaeae P7-E3, Eggerthella lenta P7-G7, Raoultibacter massiliensis P7- A2, Collinsella intestinalis P8-C1, Adlercreutzia equolifaciens P11-C8*, and *Clostridium citroniae P2-B6*. Similar to 3-oxoLCA, isoLCA inhibited Th17 cell differentiation through RORγt. Bacterial hydroxysteroid dehydrogenases convert LCA to 3-oxoLCA and isoLCA (173). **(D)** Mice fed an inulin diet showed changes in the gut microbiota and BA production associated with type 2 inflammation in the intestine and lungs, including production of IL-33 and activation of group 2 innate lymphoid cells and eosinophilia (174). **(E)** TMAO production induces NLRP3 inflammasome and caspase-1 activity leading to increased inflammation; long-term activation of these pathways contributes to obesity, CVD, and T2D, as reviewed in (15). Interestingly, while dietary TMA disrupted BBB function and tight junction integrity, TMAO enhanced BBB integrity and protected against inflammatory insult through tight junction regulator annexin A1 (175). **(F)** Generally, LPS binds TLR4 located on myeloid cells and adipocytes activating inflammatory pathways and causing disruption of the intestinal barrier as reviewed in (166, 176). However, MedDiet adherence in human studies resulted in reduced levels of circulating LPS markers, reduced inflammation, and maintained intestinal barrier integrity (158). Dashed arrows represent the outcome following blockage of LPS action. **(G)** As shown in Table 3, MedDiet alters the gut microbiota in populations with obesity, including the change in prevalence of several taxa: *Bacteroides* spp. (42, 63, 65), *Roseburia* spp. (42, 63, 91), *Lachnospira* spp. (63, 65, 91, 168), *Ruminococcus* spp. (42, 91, 137), *Akkermansia* spp. (54, 162, 167), and *Faecalibacterium prausnitzii* (42, 91, 154, 163), and generally leads to increased bacterial diversity and a reduced Firmicutes/Bacteroides ratio, which were decreased and increased with obesity, respectively, as reviewed in (11, 12). Image created with BioRender.com. 3-oxoLCA, 3-oxolithocholic acid; BAs, bile acids; BBB, blood-brain barrier; CA, cholic acid; CRP, C-reactive protein; CVD, cardiovascular disease; F/B, Firmicutes/Bacteroidetes; FXR, farnesoid X receptor; GRP, G-protein-coupled receptor; IL, interleukin; ILC2, group 2 innate lymphoid cells; isoalloLCA, isoallolithocholic acid; isoLCA, isolithocholic acid; LCA, lithocholic acid; LPS, lipopolysaccharide; MCP, monocyte chemotactic protein; MedDiet, Mediterranean diet; mitoROS, mitochondrial reactive oxygen species; MSC, mesenchymal stromal cell; NF-κB, nuclear factor kappa-light-chain-enhancer of activated B cells; NLRP3, NOD-like receptor family pyrin domain-containing 3; RORγt, retinoid-related orphan receptor γt; SCFAs, short-chain fatty acids; T2D, type-2 diabetes; Th17, T helper cells expressing IL-17; TLR4, toll-like receptor 4; TMA, trimethylamine; TMAO, trimethylamine N-oxide; TNF-α, tumor necrosis factor alpha; Treg, regulatory T cells.

SCFAs are derived from the fermentation of non-digestible dietary fiber by gut bacteria, and they play a critical role in intestinal physiology. Acetate, propionate, and butyrate account for 95% of the SCFAs in the intestinal tract. In a healthy individual, < 5% of SCFAs are excreted in feces, as most are absorbed through the gut mucosa and utilized in the gut, while some enters the bloodstream (12). While dietary fiber has been shown to promote weight loss and improve glycemic control, the complete biological role of SCFAs in this process remains unclear (83). Some SCFAs have been shown to beneficially affect host metabolism through secretion of gut hormones, such as glucagon-like peptide-1 (GLP1) and peptide YY, that affect appetite, reduce inflammation, and increase fat oxidation, as reviewed by Hernandez et al. (169). Studies in mice, which allow experimental designs that cannot be performed in humans, have added greatly to our understanding of diet contributions to gut microbiota-derived metabolic changes. Recently, Bachem et al. showed the impact of gut microbiota on the fate of CD8^+^ T-cells through the production of SCFAs in mice consuming a high-fiber diet (Figure 1) (171). SCFA supplementation also restored the number of enteric neurons that were depleted following antibiotic treatment in mice (177). In mice, reduction of SCFAs by a fiber-deficient diet led to alterations of the gut microbiota, increased intestinal permeability, inflammation, and cognitive impairment. Furthermore, SCFA supplementation improved these deficits (178). These recent studies support a role for SCFAs in modulation of immunity, inflammation, and potentially obesity.

Dietary fiber that affects production of SCFAs can come from many sources, but mostly fruits, vegetables, and whole grains, which are staples of a MedDiet. Several recent clinical studies have shown that there is increased production of some SCFAs, based on blood and/or feces measurements (see Table 2), following MedDiet intervention in healthy volunteers (66) or individuals with disorders such as ulcerative colitis (63), intestinal barrier impairment (158), overweight, or obesity (90, 167). Observational studies have also shown that better adherence to the MedDiet has been associated with increased levels of SCFAs (128, 132, 144). Another study of individuals who followed a hypocaloric MedDiet (*n* = 21), a very-low-calorie ketogenic diet (*n* = 18) and volunteers who underwent sleeve gastrectomy bariatric surgery (*n* = 22) showed MedDiet was enriched in several pathways related to SCFA fermentation (179). Following MedDiet intervention in volunteers with overweight, SCFAs were negatively correlated with changes in some inflammatory cytokines, including VEGF, MCP-1, IL-17, IP-10, and IL-12 (90). Meanwhile, a few studies have shown no significant changes (91) or decreases (65) in SCFAs following a MedDiet. Tracking the changes in specific SCFAs may be useful to our understanding as future studies continue.

Due to resource constraints, many intervention studies are limited to a very short time interval with the longest dietary intervention investigating changes in SCFAs described above spanning 3 months. This short time frame sets a significant limitation on the conclusions that can be drawn from the studies in terms of generalizing to a stable effect over years. In a small study of 21 healthy-weight individuals who consumed a MedDiet for 3 days, then a Canadian diet [reflecting the average Canadian dietary intake, which would be considered a Western diet (180)] for 13 days, followed by a MedDiet for an additional 3 days, circulating SCFAs and branched-chain fatty acids were not significantly altered by the first MedDiet intervention, but propionate, valerate, isobutyrate, and isovalerate were increased by the Canadian diet, then decreased after the second MedDiet (65). While the data show that circulating SCFA concentrations can be altered following MedDiet, and changes can occur over a short period of time, one must be careful about extrapolation of results from a few days to an effect that might occur after years of following a specific dietary pattern. Additional interventional long-term studies are needed to resolve discrepancies between study results and to assess whether these changes remain stable over time.

A systemic meta-analysis of 34 animal studies showed that diets rich in anthocyanin-rich fruits and vegetables significantly reduced the Firmicutes/Bacteroides ratio and increased SCFA production in rodents. They found that higher production of acetic acid, butanoic acid, and propionic acid was observed with longer periods of dietary intervention (≥4 weeks) and higher doses of anthocyanins. Anthocyanin supplementation had the greatest effect on acetic acid concentration in high fat/cholesterol diet models, while the greatest effect on butanoic acid and propionic acid were in HFD-induced obesity models (181). These studies provide an initial understanding of the role of specific components of the MedDiet in modulating the gut microbiota and gut-derived SCFAs.

Because humans with obesity have been reported to display excessive levels of fecal SCFAs (potentially due to lack of ability to metabolize and absorb these metabolites), as reviewed in (11, 112, 182), a strategy was proposed to combat obesity by altering the gut microbiota with a goal to modulate the number of SCFA-metabolizing or SCFA-producing bacteria through a dietary change. In a small study of 20 elderly women, obesity was associated with an increase in pro-inflammatory *Collinsella* spp. and *Streptococcus* spp. There was also a decrease in SCFA-producers, including *Lachnospiraceae* and *Ruminococcaceae*. Relative abundance of *Collinsella* spp. was reduced following both a hypocaloric MedDiet for 15 days and a hypocaloric MedDiet enriched with a probiotic mixture for 15 days (and both included an individual-based exercise regimen) (88). These studies make associations between MedDiet, SCFA, inflammation, and obesity.

In a sub-study of the PREDIMED trial in volunteers with overweight/obesity, an energy-restricted MedDiet resulted in weight loss and changes in the gut microbiota after a 1-year intervention. While SCFAs were not measured directly, there was an increase in some SCFA-producing microbes, including *Lachnospira* spp. and *Lachnospiraceae NK4A136* (57). When selecting subjects with the highest fecal butyrate increase at 4 weeks after MedDiet initiation, higher relative abundances of *Faecalibacterium prausnitzii* and *Lachnospiraceae* family were also observed (91). Likewise, the Obekit study found SCFA-producing bacteria, including *Bifidobacterium animalis, Oscillibacter valericigenes, Oscillospira (Flavonifractor) plautii, Ruminococcus bromii, Roseburia faecis*, and *Paraprevotella clara*, in a northern-Spanish population with overweight/obesity and high MedDiet adherence (89). While our discussion is focused on the MedDiet, many studies have combined caloric restriction with the MedDiet, so interpretation of results cannot distinguish the contribution of each of these two variables in many cases. However, it is evident that diets rich in fiber, flavonoids, and polyphenols, such as the MedDiet, are shown to impact the gut microbiota composition, and importantly, increase the number of bacteria with the ability to produce and metabolize SCFAs. The studies summarized in Table 2 report circulating and/or fecal SCFA levels. One cautionary note derives from a study by Farhat et al. that demonstrated poor correlation between serum and fecal SCFAs, concluding that one is not a good proxy for the other (183). In summary, many studies associate a MedDiet with an increase in SCFAs, and mechanisms are proposed by which SCFAs modulate immunity/inflammation; however, results are not entirely consistent across studies, so work is ongoing.

### 5.2 Bile acids

BAs, which are secreted into the intestine in the presence of fats as part of the digestive process, are generated from dietary lipids, cholesterol, and fat-soluble vitamins in hepatocytes via two main synthetic pathways. Primary BAs are stored in the gallbladder and secreted in the gut after conjugation. Secondary BAs are generated via further interaction of primary BAs with the gut microbiome. Similar to SCFAs, BAs are critically important in gut physiology, and secondary BAs alter the gene expression of enterocytes and of gut bacteria (184). Elevated secondary BAs in serum and feces have been associated with increased inflammation. Secondary BAs act as ligands for G-protein-coupled bile acid receptor 1 and farnesoid X receptor (FXR), the activation of which mediates immunity and promotes anti-inflammatory effects. Under normal conditions, there is a balance between primary and secondary BAs; however, this balance can be disrupted by gut microbiota dysbiosis (185).

While some mechanisms of BA metabolism are known and can be reviewed here (186), others have yet to be explored. Hang et al. showed that derivatives of the secondary BA lithocholic acid (LCA), mediate host immune response by mediating T helper cells expressing IL-17A (T_H_17) and regulatory T (Treg) cell differentiation. The metabolites 3-oxoLCA and isoLCA were shown to inhibit T_H_17 cell differentiation by binding to transcription factor retinoid-related orphan receptor (ROR) γt (172, 173), while production of mitochondrial reactive oxygen species by isoalloLCA increased expression of FoxP3 and Treg cell differentiation (172) (Figure 1). A diet of inulin fiber altered the composition of mouse microbiota and lead to increased production of BAs, which is presumed to have aided in the production of IL-33 and activation of innate lymphoid cells and eosinophils to promote type 2 inflammation (Figure 1). These affects were BA-dependent because (1) depletion of the BA receptor FXR and (2) genetic deletion of a BA-metabolizing enzyme abrogated these affects (174). HFD-fed mice with obesity had significantly increased taurine-conjugated BAs, but these affects were nearly abrogated in NLRP3-deficiency (81). During the last several years, there have been significant discoveries regarding the role of BAs in immunity and inflammation; it is proposed that manipulation of the gut microbiota, and thus of BA production, may be a useful approach to treatment for obesity.

There is an interdependent relationship between the host biological pathways and bacterial metabolism. The gut microbiome has been shown to impact the chemistry of all organs, including amino acid conjugations of host BAs (186). Conversely, BAs have considerable effects on the structure of the gut microbial community; they can stimulate the growth of microbes that utilize BAs as an energy source and repress the growth of microbes that are intolerant of its effects (184). A recent study suggested that human gut bacteria from many families within the Actinobacteria and Firmicutes phyla produce 3-oxoLCA, including *Gordonibacter pamelaeae* P7-E3, *Eggerthella lenta* P7-G7, *Raoultibacter massiliensis* P7- A2, *Collinsella intestinalis* P8-C1, *Adlercreutzia equolifaciens* P11-C8, and *Clostridium citroniae* P2-B6 and these may work together to affect the immune system (173).

In a fecal microbiota transplant pilot clinical trial in volunteers with obesity, BA profiles were modified to match that of the lean donor after 12 weeks, including sustained reduction in taurocholic acid, without any change in BMI (104). This trial did not document any change in BMI, however the 12-week time period may not have been long enough to capture significant weight change and future studies with a longer trial period are needed. A secondary analysis from fecal samples collected from these volunteers identified *Bacteroides ovatus* and *Phocaeicola dorei*, which positively correlated with unconjugated BAs, and *Bifidobacterium adolescentis, Collinsella aerofaciens*, and *Faecalibacterium prausnitzii*, which positively correlated with secondary BAs, as the bacterial species candidates that affected gut BA metabolism (187). In addition to dietary components, the caloric level of a diet must be considered, as calorie restriction has been shown to decrease production of BAs (188, 189). Supplementation with non-12α-hydroxylated BAs in mice increased thermogenesis and slowed weight gain (188). It is well-known that BAs impact the gut microbiota and are influenced by dietary changes, however, there are limited studies that have incorporated the measurement of BAs in relation to the MedDiet pattern.

The MedDiet pattern limits the amount and types of dietary fat intake, and therefore has potential to change the amounts and types of BAs produced by the host. In MedDiet intervention studies, lower production of primary and secondary BAs has been observed (42, 91). Fecal secondary BAs were significantly reduced by 4 weeks and primary BAs were reduced by 8 weeks following initiation of a MedDiet. Volunteers with the greatest reduction of total BAs also had higher baseline levels of *Bilophila wadsworthia*, which significantly decreased after 4 weeks (91). Although circulating BA levels remained unchanged after just 4 days of a MedDiet compared to a fast-food diet, the primary to secondary BA ratio was found to positively correlate with *Bifidobacterium* spp. and negatively correlate with *Roseburia* spp. (168).

Dietary diversity has been shown to inversely correlate with several circulating secondary BAs (190), and a MedDiet tends to have more diverse foods than a Western diet (191). A variety of fruits and vegetables eaten on a MedDiet contain flavonoids, which are shown to have anti-inflammatory properties, in part through pathways involving BAs. In murine studies, administration of the hops-derived prenylated flavonoid xanthohumol, and its semi-synthetic derivative tetrahydroxanthohumol, altered the gut microbiota and BA metabolism, and reduced adipose tissue inflammation (192). The MedDiet also promotes intake of whole grains compared to refined grains, as eaten in the typical Western diet. Two secondary BAs were lowest in a diet of unrefined carbohydrates composed from a high proportion of whole grain foods (193). A single fat source alone may not be enough to alter BA production in humans, as consumption of virgin olive oil with or without thyme did not alter BA production when volunteers were asked to limit their polyphenol-rich food intake (96). Compared to Western diet, MedDiet promotes reduced overall fat intake, with primary intake of healthy fats like olive oil, resulting in lowered production of secondary BAs, favoring reduced inflammation and a decreased risk of obesity.

### 5.3 TMAO and its dietary precursors

TMAO is a product of choline, L-carnitine, betaine, and ergothioneine via metabolism by the gut microbiota. Trimethylamine is generated within the intestinal lumen by enzymatic changes of the aforementioned precursors, absorbed from the intestine, and transferred to the liver where flavin-dependent monooxygenase isoforms 1 and 3 convert it to TMAO. Dietary choline and L-carnitine are primarily found in animal products while betaine is mostly from plants. Dietary ergothioneine is found in both some animal (mostly liver and kidney organs) and plant (including mushrooms and beans) products (83, 194). Krueger et al. describe the negative effects of elevated TMAO on adipose tissue as it relates to the discussion of obesity (108). Increases in TMAO has been found to correlate with an increase in BMI and visceral adipose and TMAO levels over 8.2 μM predict the occurrence metabolic syndrome (77). Obese mice that had a TMAO-producing enzyme (FMO3) conferred protection against obesity (109). TMAO also have an inflammatory effect through activation of the NLRP3 inflammasome (195). However, similar to other metabolites, the physiology of TMAO is complex, as TMAO acts on multiple organs, with evidence of beneficial effects in the brain. Long-term exposure to TMAO in mice protected the brain from inflammatory challenge with LPS and reduced activation of astrocytes and microglia (Figure 1) (175). While some studies might paint TMAO as a negative factor and many proposed healthy diets eliminate or strictly limit red meat, the study by Hoyles *et al*. suggest that the full picture of TMAO's role in obesity and other metabolic syndromes has yet to be understood.

Elimination of red meats, which are known to be a source of dietary choline, is encouraged on the MedDiet. An observational study comparing healthy adults found circulating levels of TMAO negatively correlated with MedDiet score after adjusting for BMI, physical activity, and total energy intake (123). A similar result was seen in a separate study with a population of volunteers including 30% with obesity, as those with a morning chronotype (a term used to describe a person's circadian preferences) had the highest adherence to a MedDiet and the lowest circulating TMAO concentrations (125, 127). Choline participates in multiple essential functions, including serving as a precursor of essential cellular components, and is oxidized to betaine in the methylation cycle of multiple pathways.

A lower betaine/choline (B/C) ratio is associated with features of metabolic syndrome, so the B/C ratio is a biomarker of metabolic function. A case-cohort study within the PREDIMED trial found that volunteers assigned to the MedDiet intervention with a high B/C ratio had a lower risk of CVD compared to controls with a low B/C ratio (136). In contrast, a separate case-cohort of the PREDIMED study, found that individuals with the highest quartile of baseline TMAO and α-glycerophosphocholine had a lower risk of T2D (196). One-year follow-up data from the Spanish PREDIMED-Plus trial showed the greatest increase in dietary choline or betaine intake was associated with improved serum glucose and HbA1c levels, as well as reduced body weight and total cholesterol in subjects with overweight/obesity (197). A secondary analysis of a randomized clinical trial in adults with overweight/obesity comparing the effects of consuming different concentrations of unprocessed lean red meat, along with a MedDiet, found that lower consumption of red meat resulted in lower serum TMAO concentrations after 5 weeks (141). In individuals with healthy weight, there was a 1.5-fold increase in urinary TMAO after 2 weeks of a MedDiet compared to a Western-type diet (87). However, another study involving adults with overweight/obesity found urinary carnitine was significantly reduced by 4 weeks following MedDiet intervention and remained reduced at 8 weeks (91).

Few studies have examined associations between TMAO, MedDiet, and gut microbiome composition. *Prevotella copri* was significantly lower in female non-human primates fed a MedDiet compared to a Western Diet. Interestingly, among those fed a Western-diet, those who had highest amounts of *P. copri* also had elevated levels of urinary carnitine-based metabolites (18). Another study found no difference in plasma TMAO levels, or its precursors, before and after a 6-month MedDiet intervention in healthy adults at risk of colon cancer. However, the relative abundance of *Akkermansia mucinophilia* in colon biopsies was modestly and inversely associated with TMAO, betaine, choline, and carnitine at baseline, and this association was weaker at 6 months following MedDiet introduction (134).

A study involving children and adolescents with obesity found that non-responders (defined as subjects whose BMI z-score was maintained or increased) to nutritional or exercise regimens had significantly increased choline and a decreased B/C ratio after 6 months. Increased choline was associated with *Romboutsia timonensis, Granulicatella adiacens*, and *Aminipila butyrica*, while decreased choline was associated with *Enterocloster aldensis*. *Anaerotignum faecicola* and *Bacteroides stercoris* were associated with a decreased B/C ratio. Volunteers with both increased choline and a decreased B/C ratio had higher abundance of *Romboutsia timonensis, Granulicatella adiacens*, and *Pediococcus stilesii* (198). While it is exciting to see specific bacterial species identified as playing a role in TMAO biology, future RCTs that examine the effects of MedDiet on TMAO are required to draw conclusions on this topic.

### 5.4 Lipopolysaccharide

LPS is also called endotoxin and is derived from Gram-negative bacterial membranes. Previous studies have shown trends toward lower endotoxemia in association with Mediterranean-like diets, while Western-style diets are associated with increased endotoxemia (149, 199, 200). A mechanism by which the MedDiet may contribute to improved metabolic health is through the modulation of the gut microbiota which can lead to a reduction of metabolic endotoxemia (184). We are beginning to discover some of the detailed physiology of LPS action. LPS has also been shown to correlate with the incidence of cardiovascular events, potentially through upregulation of proprotein convertase subtilisin/kexin type 9 involved in a mechanism associated with NADPH oxidase (Nox2)-related oxidative stress (150). Yogurt supplementation, with associated probiotic bacteria, attenuated metabolic endotoxemia and inflammation in mice with obesity likely through reduced activation of the TLR4 signaling pathway (201). In mice, HFD significantly increased levels of LPS binding protein (LBP). However, depletion of the NLRP3 inflammasome using knock-out genotyping abrogated the levels of LBP, implicating the NLRP3 inflammasome as a target to mediate obesity-related inflammation (81).

The MedDiet is rich in polyphenols from various foods such as berries, spices, nuts, cocoa, wine, and olive oil, among others. Polyphenol-rich diets have beneficial effects against obesity-related dysbiosis and circulating LPS levels. For example, isoflavones showed reduced production of nitric oxide species and reduced pro-inflammatory cytokine (TNF-α and IL-6) release in response to LPS (202). In a cross-sectional study of older adults (60% with overweight or obesity) greater adherence to Mediterranean-like diets (MedDiet and prudent diet) were associated with lower circulating 3-hydroxy fatty acids levels, a proxy of LPS burden (121). As part of the Progression of Liver Disease and Cardiovascular Disorders in Non-alcoholic Fatty Liver disease (PLINIO) study, soluble Nox2-derived peptide, a marker of systemic oxidative stress, and serum LPS, were higher in patients with overweight/obesity and correlated with low adherence to MedDiet (41). In the LIBRE study, women with intestinal barrier impairment were allocated to follow MedDiet (n=124) or a control diet (*n* = 136) for 3 months. Adherence to MedDiet was associated with decreased LBP and gut permeability (Figure 1) (158). While circulating LPS is typically enhanced in obesity, the MedDiet may help lessen these levels and reduce inflammation.

Olive oil is a critical component of the MedDiet and it has been shown to be protective against inflammation. Virgin olive oil phenolic extract was protective against LPS treatment in murine and human brain cells by reducing activation of TLR4 and the NLRP3 signaling cascade (80). Olive oil consumption was also associated with a less significant increase in blood glucose, a more marked increase in blood insulin and GLP1, and a significant reduction in LPS and gut permeability, in individuals with impaired fasting glucose (203). These recent studies summarize the ability of the MedDiet to reduce the risk of obesity by maintaining intestinal barrier integrity and reducing the amount of circulating LPS, thus reducing the associated inflammation.

## 6 Conclusions and future directions

Diet has a major impact on obesity and the composition of the gut microbiota, and in turn, the types of microbial metabolites in the gut. While some studies suggest that diet may be a key component of an effective treatment for obesity and for the restoration of homeostasis (11), a recent systemic review examining the effects of the MedDiet on the gut microbiota and gut metabolites found inconsistent and few significant changes, which may be attributed to differences in methods, cohort characteristics, and study quality (86). For example, scales to evaluate the MedDiet used in the studies we reviewed range from 8- to 24-point scores, thus emphasizing different dietary components (Table 1). The populations evaluated in each study also varied, from healthy adults (65, 77, 128), to older adults (42, 121, 154), to individuals with health conditions such as ulcerative colitis (55, 63) and MASLD (41). While adherence to the MedDiet improves health and has some effect on the gut microbiome, more work needs to be done to determine the extent to which the MedDiet-associated changes in gut microbiota and their metabolites mediate these health-promoting effects. Two other critical elements important in assessing the risk of obesity are an individual's physical activity level and the total calories consumed per day, so examination of diet patterns without controlling for both exercise and caloric intake, and other potential confounders, may contribute to the conflicting results for various studies.

In a recent review, Gundogdu and Nalbantoglu note the mixed results may be due to lack of standardization, study design limitations, and differences in the defined MedDiet (204). Though widely accepted, amplicon sequencing approaches (i.e., 16S ribosomal RNA gene sequencing) lack the depth to capture most strain-specific microbes and their functionality (43, 204). Additionally, an individual's specific dietary preferences and responses, whether from a baseline diet or self-selected food during an intervention, also affect gut microbiota composition, as reviewed in Fassarella et al. (205), and further complicate adequately controlled studies. Given the number of potentially confounding variables, rigorous studies that incorporate measuring or restricting as many of these variables as possible, in addition to sequencing of the gut microbiota and measuring metabolites, are needed.

Obesity is a growing public health concern, and a better understanding of the pathophysiology related to diet and lifestyle is needed. Harnessing the ability to systematically change the gut microbiota, and correspondingly change the microbial metabolites, is a therapeutic target for investigators and clinicians. However, larger and more rigorous clinical trials are needed, in addition to animal studies that can decode the mechanisms, to define the pathophysiology of obesity. This will be challenging given the number of interacting parts between the gut microbiota, metabolites, and host immunity. While unsettling to consider the huge task of dissecting the complex biology of dietary nutrients, inflammation, and health consequences, tremendous progress has been made in the past few decades. The heterogeneity between studies is not surprising given the number of potential confounding variables; nevertheless, there is clear evidence to support the benefits of a Mediterranean-like dietary pattern as a means to alter the gut microbiota, gut metabolites, and essential biological pathways within populations with overweight/obesity.

## Author contributions

MF: Writing – original draft. EA: Writing – original draft. KF: Writing – review & editing, Conceptualization. AB: Writing – review & editing, Writing – original draft, Conceptualization.

## Footnotes

^1^World Health Organization. Available online at: https://www.who.int/news-room/fact-sheets/detail/obesity-and-overweight (accessed on January 11, 2024).

## References

[1] Chew NWS Ng CH Tan DJH Kong G Lin C Chin YH. The global burden of metabolic disease: Data from 2000 to 2019. Cell Metab. (2023) 35:414–28 e3. 10.1016/j.cmet.2023.02.003 36889281

[2] GBD 2019 Diseases and Injuries Collaborators. Global burden of 369 diseases and injuries in 204 countries and territories, 1990-2019: a systematic analysis for the Global Burden of Disease Study 2019. Lancet. (2020) 396:1204–22. 10.1016/S0140-6736(20)30925-9 33069326 PMC7567026

[3] St-Onge MP Heymsfield SB. Overweight and obesity status are linked to lower life expectancy. Nutr Rev. (2003) 61:313–6. 10.1301/nr.2003.sept.313-316 14552067

[4] Masood B Moorthy M. Causes of obesity: a review. Clin Med. (2023) 23:284–91. 10.7861/clinmed.2023-0168 37524429 PMC10541056

[5] Mukherjee MS Han CY Sukumaran S Delaney CL Miller MD. Effect of anti-inflammatory diets on inflammation markers in adult human populations: a systematic review of randomized controlled trials. Nutr Rev. (2022) 81:55–74. 10.1093/nutrit/nuac045 35831971

[6] Li Q Wang Q Xu W Ma Y Wang Q Eatman D. C-reactive protein causes adult-onset obesity through chronic inflammatory mechanism. Front Cell Dev Biol. (2020) 8:18. 10.3389/fcell.2020.00018 32154244 PMC7044181

[7] Kawai T Autieri MV Scalia R. Adipose tissue inflammation and metabolic dysfunction in obesity. Am J Physiol Cell Physiol. (2021) 320:C375–91. 10.1152/ajpcell.00379.2020 33356944 PMC8294624

[8] Calder PC Ahluwalia N Brouns F Buetler T Clement K Cunningham K. Dietary factors and low-grade inflammation in relation to overweight and obesity. Br J Nutr. (2011) 106 Suppl 3:S5–78. 10.1017/S0007114511005460 22133051

[9] Wang L Gao T Li Y Xie Y Zeng S Tai C. A long-term anti-inflammation markedly alleviated high-fat diet-induced obesity by repeated administrations of overexpressing IL10 human umbilical cord-derived mesenchymal stromal cells. Stem Cell Res Ther. (2022) 13:259. 10.1186/s13287-022-02935-8 35715850 PMC9204983

[10] Muscogiuri G Verde L Sulu C Katsiki N Hassapidou M Frias-Toral E. Mediterranean diet and obesity-related disorders: what is the evidence? Curr Obes Rep. (2022) 11:287–304. 10.1007/s13679-022-00481-1 36178601 PMC9729142

[11] Lange O Proczko-Stepaniak M Mika A. Short-chain fatty acids-A product of the microbiome and its participation in two-way communication on the microbiome-host mammal line. Curr Obes Rep. (2023) 12:108–26. 10.1007/s13679-023-00503-6 37208544 PMC10250490

[12] You H Tan Y Yu D Qiu S Bai Y He J. The therapeutic effect of SCFA-mediated regulation of the intestinal environment on obesity. Front Nutr. (2022) 9:886902. 10.3389/fnut.2022.886902 35662937 PMC9157426

[13] Agus A Clement K Sokol H. Gut microbiota-derived metabolites as central regulators in metabolic disorders. Gut. (2021) 70:1174–82. 10.1136/gutjnl-2020-323071 33272977 PMC8108286

[14] Sipe LM Chaib M Pingili AK Pierre JF Makowski L. Microbiome, bile acids, and obesity: How microbially modified metabolites shape anti-tumor immunity. Immunol Rev. (2020) 295:220–39. 10.1111/imr.12856 32320071 PMC7841960

[15] Grosso G Laudisio D Frias-Toral E Barrea L Muscogiuri G Savastano S Colao A. Anti-Inflammatory Nutrients And Obesity-Associated Metabolic-Inflammation: State Of The Art And Future Direction. Nutrients. (2022) 14:1137. 10.3390/nu14061137 35334794 PMC8954840

[16] Lecube A Lopez-Cano C. Obesity, a diet-induced inflammatory disease. Nutrients. (2019) 11:2284. 10.3390/nu11102284 31554199 PMC6835563

[17] Calabrese FM Porrelli A Vacca M Comte B Nimptsch K Pinart M. Metaproteomics approach and pathway modulation in obesity and diabetes: a narrative review. Nutrients. (2021) 14:47. 10.3390/nu14010047 35010920 PMC8746330

[18] Newman TM Shively CA Register TC Appt SE Yadav H Colwell RR. Diet, obesity, and the gut microbiome as determinants modulating metabolic outcomes in a non-human primate model. Microbiome. (2021) 9:100. 10.1186/s40168-021-01069-y 33952353 PMC8101030

[19] Rogero MM Calder PC. Obesity, inflammation, toll-like receptor 4 and fatty acids. Nutrients. (2018) 10:432. 10.3390/nu10040432 29601492 PMC5946217

[20] Zhao Y Lin J Fan Y Lin X. Life cycle of Cryptococcus neoformans. Annu Rev Microbiol. (2019) 73:17–42. 10.1146/annurev-micro-020518-120210 31082304 PMC12860491

[21] Shivappa N Steck SE Hurley TG Hussey JR Hebert JR. Designing and developing a literature-derived, population-based dietary inflammatory index. Public Health Nutr. (2014) 17:1689–96. 10.1017/S1368980013002115 23941862 PMC3925198

[22] Hodge AM Karim MN Hebert JR Shivappa N Milne RL de Courten B. Diet scores and prediction of general and abdominal obesity in the Melbourne collaborative cohort study. Public Health Nutr. (2021) 24:6157–68. 10.1017/S1368980021001713 33875030 PMC11148580

[23] May KS den Hartigh LJ. Gut microbial-derived short chain fatty acids: impact on adipose tissue physiology. Nutrients. (2023) 15:272. 10.3390/nu15020272 36678142 PMC9865590

[24] Las Heras V Melgar S MacSharry J Gahan CGM. The influence of the Western diet on microbiota and gastrointestinal immunity. Annu Rev Food Sci Technol. (2022) 13:489–512. 10.1146/annurev-food-052720-011032 34990225

[25] Khan S Luck H Winer S Winer DA. Emerging concepts in intestinal immune control of obesity-related metabolic disease. Nat Commun. (2021) 12:2598. 10.1038/s41467-021-22727-7 33972511 PMC8110751

[26] Guasch-Ferre M Willett WC. The Mediterranean diet and health: a comprehensive overview. J Intern Med. (2021) 290:549–66. 10.1111/joim.13333 34423871

[27] Willett WC Sacks F Trichopoulou A Drescher G Ferro-Luzzi A Helsing E. Mediterranean diet pyramid: a cultural model for healthy eating. Am J Clin Nutr. (1995) 61:1402S−6S. 10.1093/ajcn/61.6.1402S 7754995

[28] United States Department of Agriculture. Available online at: https://www.dietaryguidelines.gov/resources/2020-2025-dietary-guidelines-online-materials (accessed August 1, 2024).

[29] Davis C Bryan J Hodgson J Murphy K. Definition of the Mediterranean diet; a literature review. Nutrients. (2015) 7:9139–53. 10.3390/nu7115459 26556369 PMC4663587

[30] Itsiopoulos C Mayr HL Thomas CJ. The anti-inflammatory effects of a Mediterranean diet: a review. Curr Opin Clin Nutr Metab Care. (2022) 25:415–22. 10.1097/MCO.0000000000000872 36039924

[31] Zupo R Castellana F Piscitelli P Crupi P Desantis A Greco E. Scientific evidence supporting the newly developed one-health labeling tool “Med-Index”: an umbrella systematic review on health benefits of mediterranean diet principles and adherence in a planeterranean perspective. J Transl Med. (2023) 21:755. 10.1186/s12967-023-04618-1 37885010 PMC10601192

[32] Mambrini SP Menichetti F Ravella S Pellizzari M De Amicis R Foppiani A. Ultra-processed food consumption and incidence of obesity and cardiometabolic risk factors in adults: a systematic review of prospective studies. Nutrients. (2023) 15:25883. 10.3390/nu15112583 37299546 PMC10255607

[33] Schlesinger S Neuenschwander M Schwedhelm C Hoffmann G Bechthold A Boeing H. Food groups and risk of overweight, obesity, and weight gain: a systematic review and dose-response meta-analysis of prospective studies. Adv Nutr. (2019) 10:205–18. 10.1093/advances/nmy092 30801613 PMC6416048

[34] Pagliai G Dinu M Madarena MP Bonaccio M Iacoviello L Sofi F. Consumption of ultra-processed foods and health status: a systematic review and meta-analysis. Br J Nutr. (2021) 125:308–18. 10.1017/S0007114520002688 32792031 PMC7844609

[35] Deledda A Palmas V Heidrich V Fosci M Lombardo M Cambarau G. Dynamics of gut microbiota and clinical variables after ketogenic and Mediterranean diets in drug-naive patients with type 2 diabetes mellitus and obesity. Metabolites. (2022) 12:1092. 10.3390/metabo12111092 36355175 PMC9693465

[36] Di Rosa C Lattanzi G Spiezia C Imperia E Piccirilli S Beato I. Mediterranean diet versus very low-calorie ketogenic diet: effects of reaching 5% body weight loss on body composition in subjects with overweight and with obesity-a cohort study. Int J Environ Res Public Health. (2022) 19:13040. 10.3390/ijerph192013040 36293616 PMC9603454

[37] Gioxari A Grammatikopoulou MG Katsarou C Panagiotakos DB Toutouza M Kavouras SA. A modified Mediterranean diet improves fasting and postprandial glucoregulation in adults with overweight and obesity: a pilot study. Int J Environ Res Public Health. (2022) 19:215347. 10.3390/ijerph192215347 36430066 PMC9692994

[38] Jospe MR Roy M Brown RC Haszard JJ Meredith-Jones K Fangupo LJ. Intermittent fasting, Paleolithic, or Mediterranean diets in the real world: exploratory secondary analyses of a weight-loss trial that included choice of diet and exercise. Am J Clin Nutr. (2020) 111:503–14. 10.1093/ajcn/nqz330 31879752

[39] Hodge AM Karim MN Hébert JR Shivappa N de Courten B. Association between diet quality indices and incidence of type 2 diabetes in the Melbourne collaborative cohort study. Nutrients. (2021) 13:114162. 10.3390/nu13114162 34836416 PMC8622769

[40] Hoofnagle JH Doo E. Letter to the editor: a multi-society Delphi consensus statement on new fatty liver disease nomenclature. Hepatology. (2024) 79:E91–E2. 10.1097/HEP.0000000000000695 37983836

[41] Baratta F Pastori D Bartimoccia S Cammisotto V Cocomello N Colantoni A. Poor adherence to Mediterranean diet and serum lipopolysaccharide are associated with oxidative stress in patients with non-alcoholic fatty liver disease. Nutrients. (2020) 12:1732. 10.3390/nu12061732 32531941 PMC7352324

[42] Ghosh TS Rampelli S Jeffery IB Santoro A Neto M Capri M. Mediterranean diet intervention alters the gut microbiome in older people reducing frailty and improving health status: the NU-AGE 1-year dietary intervention across five European countries. Gut. (2020) 69:1218–28. 10.1136/gutjnl-2019-319654 32066625 PMC7306987

[43] Wang DD Nguyen LH Li Y Yan Y Ma W Rinott E. The gut microbiome modulates the protective association between a Mediterranean diet and cardiometabolic disease risk. Nat Med. (2021) 27:333–43. 10.1038/s41591-020-01223-3 33574608 PMC8186452

[44] Magriplis E Panagiotakos D Kyrou I Tsioufis C Mitsopoulou AV Karageorgou D. Presence of hypertension is reduced by Mediterranean diet adherence in all individuals with a more pronounced effect in the obese: The Hellenic National Nutrition and Health Survey (HNNHS). Nutrients. (2020) 12:853. 10.3390/nu12030853 32209978 PMC7146360

[45] Sood S Feehan J Itsiopoulos C Wilson K Plebanski M Scott D. Higher adherence to a mediterranean diet is associated with improved insulin sensitivity and selected markers of inflammation in individuals who are overweight and obese without diabetes. Nutrients. (2022) 14:4437. 10.3390/nu14204437 36297122 PMC9608711

[46] Leone A Bertoli S Bedogni G Vignati L Pellizzari M Battezzati A. Association between Mediterranean diet and fatty liver in women with overweight and obesity. Nutrients. (2022) 14:3771. 10.3390/nu14183771 36904140 PMC10005716

[47] Golzarand M Moslehi N Mirmiran P Azizi F. Adherence to the DASH, MeDi, and MIND diet scores and the incidence of metabolically unhealthy phenotypes. Obes Res Clin Pract. (2023) 17:226–32. 10.1016/j.orcp.2023.04.001 37037714

[48] Barrea L Muscogiuri G Pugliese G de Alteriis G Colao A Savastano S. Metabolically Healthy Obesity (MHO) vs. Metabolically Unhealthy Obesity (MUO) phenotypes in PCOS: association with endocrine-metabolic profile, adherence to the Mediterranean diet, and body composition. Nutrients. (2021) 13:3925. 10.3390/nu13113925 34836180 PMC8624317

[49] Taskinen RE Hantunen S Tuomainen TP Virtanen JK. The associations between whole grain and refined grain intakes and serum C-reactive protein. Eur J Clin Nutr. (2022) 76:544–50. 10.1038/s41430-021-00996-1 34404933 PMC8993682

[50] Chai W Morimoto Y Cooney RV Franke AA Shvetsov YB Le Marchand L. Dietary red and processed meat intake and markers of adiposity and inflammation: the Multiethnic Cohort Study. J Am Coll Nutr. (2017) 36:378–85. 10.1080/07315724.2017.1318317 28628401 PMC5540319

[51] Mazidi M Kengne AP George ES Siervo M. The association of red meat intake with inflammation and circulating intermediate biomarkers of type 2 diabetes is mediated by central adiposity. Br J Nutr. (2021) 125:1043–50. 10.1017/S0007114519002149 31434580

[52] Montonen J Boeing H Fritsche A Schleicher E Joost HG Schulze MB. Consumption of red meat and whole-grain bread in relation to biomarkers of obesity, inflammation, glucose metabolism and oxidative stress. Eur J Nutr. (2013) 52:337–45. 10.1007/s00394-012-0340-6 22426755 PMC3549403

[53] Kolehmainen M Ulven SM Paananen J de Mello V Schwab U Carlberg C. Healthy Nordic diet downregulates the expression of genes involved in inflammation in subcutaneous adipose tissue in individuals with features of the metabolic syndrome. Am J Clin Nutr. (2015) 101:228–39. 10.3945/ajcn.114.092783 25527767

[54] Tagliamonte S Laiola M Ferracane R Vitale M Gallo MA Meslier V. Mediterranean diet consumption affects the endocannabinoid system in overweight and obese subjects: possible links with gut microbiome, insulin resistance and inflammation. Eur J Nutr. (2021) 60:3703–16. 10.1007/s00394-021-02538-8 33763720 PMC8437855

[55] Haskey N Estaki M Ye J Shim RK Singh S Dieleman LA. A Mediterranean diet pattern improves intestinal inflammation concomitant with reshaping of the bacteriome in ulcerative colitis: a randomized controlled trial. J Crohns Colitis. (2023). 10.1093/ecco-jcc/jjad073 37095601 PMC10637046

[56] Konieczna J Romaguera D Pereira V Fiol M Razquin C Estruch R. Longitudinal association of changes in diet with changes in body weight and waist circumference in subjects at high cardiovascular risk: the PREDIMED trial. Int J Behav Nutr Phys Act. (2019) 16:139. 10.1186/s12966-019-0893-3 31882021 PMC6935084

[57] Muralidharan J Moreno-Indias I Bullo M Lopez JV Corella D Castaner O. Effect on gut microbiota of a 1-y lifestyle intervention with Mediterranean diet compared with energy-reduced Mediterranean diet and physical activity promotion: PREDIMED-Plus Study. Am J Clin Nutr. (2021) 114:1148–58. 10.1093/ajcn/nqab150 34020445 PMC8408861

[58] Marcos-Pardo PJ Gonzalez-Galvez N Espeso-Garcia A Abelleira-Lamela T Lopez-Vivancos A Vaquero-Cristobal R. Association among adherence to the Mediterranean diet, cardiorespiratory fitness, cardiovascular, obesity, and anthropometric variables of overweight and obese middle-aged and older adults. Nutrients. (2020) 12:2750. 10.3390/nu12092750 32927609 PMC7551167

[59] Hosseini-Esfahani F Koochakpoor G Daneshpour MS Sedaghati-khayat B Mirmiran P Azizi F. Mediterranean dietary pattern adherence modify the association between FTO genetic variations and obesity phenotypes. Nutrients. (2017) 9:1064. 10.3390/nu9101064 28954439 PMC5691681

[60] Urpi-Sarda M Casas R Sacanella E Corella D Andres-Lacueva C Llorach R. The 3-year effect of the Mediterranean diet intervention on inflammatory biomarkers related to cardiovascular disease. Biomedicines. (2021) 9:862. 10.3390/biomedicines9080862 34440065 PMC8389558

[61] Casas R Sacanella E Urpi-Sarda M Corella D Castaner O Lamuela-Raventos RM. Long-term immunomodulatory effects of a Mediterranean diet in adults at high risk of cardiovascular disease in the PREvencion con DIeta MEDiterranea (PREDIMED) randomized controlled trial. J Nutr. (2016) 146:1684–93. 10.3945/jn.115.229476 27440261

[62] Konstantinidou V Covas MI Munoz-Aguayo D Khymenets O de la Torre R Saez G. *In vivo* nutrigenomic effects of virgin olive oil polyphenols within the frame of the Mediterranean diet: a randomized controlled trial. FASEB J. (2010) 24:2546–57. 10.1096/fj.09-148452 20179144

[63] Strauss JC Haskey N Ramay HR Ghosh TS Taylor LM Yousuf M. Weighted gene co-expression network analysis identifies a functional guild and metabolite cluster mediating the relationship between mucosal inflammation and adherence to the Mediterranean diet in ulcerative colitis. Int J Mol Sci. (2023) 24:7323. 10.3390/ijms24087323 37108484 PMC10138710

[64] Camilleri M Zheng T. Cannabinoids and the gastrointestinal tract. Clin Gastroenterol Hepatol. (2023) 21:3217–29. 10.1016/j.cgh.2023.07.031 37678488 PMC10872845

[65] Bourdeau-Julien I Castonguay-Paradis S Rochefort G Perron J Lamarche B Flamand N. The diet rapidly and differentially affects the gut microbiota and host lipid mediators in a healthy population. Microbiome. (2023) 11:26. 10.1186/s40168-023-01469-2 36774515 PMC9921707

[66] Forteza F Bourdeau-Julien I Nguyen GQ Guevara Agudelo FA Rochefort G Parent L. Influence of diet on acute endocannabinoidome mediator levels post exercise in active women, a crossover randomized study. Sci Rep. (2022) 12:8568. 10.1038/s41598-022-10757-0 35595747 PMC9122896

[67] Sanchez-Rodriguez E Biel-Glesson S Fernandez-Navarro JR Calleja MA Espejo-Calvo JA Gil-Extremera B. Effects of virgin olive oils differing in their bioactive compound contents on biomarkers of oxidative stress and inflammation in healthy adults: a randomized double-blind controlled trial. Nutrients. (2019) 11:561. 10.3390/nu11030561 30845690 PMC6470869

[68] Patti AM Carruba G Cicero AFG Banach M Nikolic D Giglio RV. Daily use of extra virgin olive oil with high oleocanthal concentration reduced body weight, waist circumference, alanine transaminase, inflammatory cytokines and hepatic steatosis in subjects with the metabolic syndrome: a 2-month intervention study. Metabolites. (2020) 10:392. 10.3390/metabo10100392 33023123 PMC7601817

[69] Predimed: Prevencion con Dieta Mediterranea. Predimed: 2018 [Supplement]. Available online at: http://www.predimed.es (accessed May 7, 2024).

[70] Lopez-Gil JF Garcia-Hermoso A Sotos-Prieto M Cavero-Redondo I Martinez-Vizcaino V Kales SN. Mediterranean diet-based interventions to improve anthropometric and obesity indicators in children and adolescents: a systematic review with meta-analysis of randomized controlled trials. Adv Nutr. (2023) 14:858–69. 10.1016/j.advnut.2023.04.011 37127186 PMC10334150

[71] Sanchez-Rosales AI Guadarrama-Lopez AL Gaona-Valle LS Martinez-Carrillo BE Valdes-Ramos R. The effect of dietary patterns on inflammatory biomarkers in adults with type 2 diabetes mellitus: a systematic review and meta-analysis of randomized controlled trials. Nutrients. (2022) 14:4577. 10.3390/nu14214577 36364839 PMC9654560

[72] Kavyani Z Musazadeh V Fathi S Hossein Faghfouri A Dehghan P Sarmadi B. Efficacy of the omega-3 fatty acids supplementation on inflammatory biomarkers: an umbrella meta-analysis. Int Immunopharmacol. (2022) 111:109104. 10.1016/j.intimp.2022.109104 35914448

[73] Mirabelli M Chiefari E Arcidiacono B Corigliano DM Brunetti FS Maggisano V. Mediterranean diet nutrients to turn the tide against insulin resistance and related diseases. Nutrients. (2020) 12:66. 10.3390/nu12041066 32290535 PMC7230471

[74] Moszak M Szulinska M Bogdanski P. You are what you eat-the relationship between diet, microbiota, and metabolic disorders-a review. Nutrients. (2020) 12:96. 10.3390/nu12041096 32326604 PMC7230850

[75] Sanna S van Zuydam NR Mahajan A Kurilshikov A Vich Vila A Vosa U. Causal relationships among the gut microbiome, short-chain fatty acids and metabolic diseases. Nat Genet. (2019) 51:600–5. 10.1038/s41588-019-0350-x 30778224 PMC6441384

[76] Antoniazzi L Arroyo-Olivares R Bittencourt MS Tada MT Lima I Jannes CE. Adherence to a Mediterranean diet, dyslipidemia and inflammation in familial hypercholesterolemia. Nutr Metab Cardiovasc Dis. (2021) 31:2014–22. 10.1016/j.numecd.2021.04.006 34039501

[77] Barrea L Arnone A Annunziata G Muscogiuri G Laudisio D Salzano C. Adherence to the Mediterranean diet, dietary patterns and body composition in women with Polycystic Ovary Syndrome (PCOS). Nutrients. (2019) 11:2278. 10.3390/nu11102278 31547562 PMC6836220

[78] Magno MS Moschowits E Morthen MK Beining MW Jansonius NM Hammond CJ. Greater adherence to a mediterranean diet is associated with lower C-reactive protein (CRP) levels, but not to lower odds of having dry eye disease. Ocul Surf. (2023) 30:196–203. 10.1016/j.jtos.2023.09.013 37783428

[79] Roncero-Ramos I Rangel-Zuniga OA Lopez-Moreno J Alcala-Diaz JF Perez-Martinez P Jimenez-Lucena R. Mediterranean diet, glucose homeostasis, and inflammasome genetic variants: The CORDIOPREV Study. Mol Nutr Food Res. (2018) 62:e1700960. 10.1002/mnfr.201700960 29573224

[80] Taticchi A Urbani S Albi E Servili M Codini M Traina G. *In vitro* anti-inflammatory effects of phenolic compounds from Moraiolo Virgin Olive Oil (MVOO) in brain cells via regulating the TLR4/NLRP3 axis. Molecules. (2019) 24:244523. 10.3390/molecules24244523 31835609 PMC6943687

[81] Sokolova M Yang K Hansen SH Louwe MC Kummen M Hov JER. NLRP3 inflammasome deficiency attenuates metabolic disturbances involving alterations in the gut microbial profile in mice exposed to high fat diet. Sci Rep. (2020) 10:21006. 10.1038/s41598-020-76497-1 33273482 PMC7712828

[82] Demaria TM Crepaldi LD Costa-Bartuli E Branco JR Zancan P Sola-Penna M. Once a week consumption of Western diet over twelve weeks promotes sustained insulin resistance and non-alcoholic fat liver disease in C57BL/6 J mice. Sci Rep. (2023) 13:3058. 10.1038/s41598-023-30254-2 36810903 PMC9942638

[83] Beam A Clinger E Hao L. Effect of diet and dietary components on the composition of the gut microbiota. Nutrients. (2021) 13:2795. 10.3390/nu13082795 34444955 PMC8398149

[84] David LA Maurice CF Carmody RN Gootenberg DB Button JE Wolfe BE. Diet rapidly and reproducibly alters the human gut microbiome. Nature. (2014) 505:559–63. 10.1038/nature12820 24336217 PMC3957428

[85] Klimenko NS Tyakht AV Popenko AS Vasiliev AS Altukhov IA Ischenko DS. Microbiome responses to an uncontrolled short-term diet intervention in the frame of the citizen science project. Nutrients. (2018) 10:576. 10.3390/nu10050576 29738477 PMC5986456

[86] Kimble R Gouinguenet P Ashor A Stewart C Deighton K Matu J. Effects of a mediterranean diet on the gut microbiota and microbial metabolites: a systematic review of randomized controlled trials and observational studies. Crit Rev Food Sci Nutr. (2022) 63:1–22. 10.1080/10408398.2022.2057416 35361035

[87] Barber C Mego M Sabater C Vallejo F Bendezu RA Masihy M. Differential effects of Western and Mediterranean-type diets on gut microbiota: a metagenomics and metabolomics approach. Nutrients. (2021) 13:2638. 10.3390/nu13082638 34444797 PMC8400818

[88] Cancello R Turroni S Rampelli S Cattaldo S Candela M Cattani L. Effect of short-term dietary intervention and probiotic mix supplementation on the gut microbiota of elderly obese women. Nutrients. (2019) 11:3011. 10.3390/nu11123011 31835452 PMC6950529

[89] Roses C Cuevas-Sierra A Quintana S Riezu-Boj JI Martinez JA Milagro FI Barcelo A. Gut microbiota bacterial species associated with mediterranean diet-related food groups in a Northern Spanish Population. Nutrients. (2021) 13:636. 10.3390/nu13020636 33669303 PMC7920039

[90] Pagliai G Russo E Niccolai E Dinu M Di Pilato V Magrini A. Influence of a 3-month low-calorie Mediterranean diet compared to the vegetarian diet on human gut microbiota and SCFA: the CARDIVEG Study. Eur J Nutr. (2020) 59:2011–24. 10.1007/s00394-019-02050-0 31292752

[91] Meslier V Laiola M Roager HM De Filippis F Roume H Quinquis B. Mediterranean diet intervention in overweight and obese subjects lowers plasma cholesterol and causes changes in the gut microbiome and metabolome independently of energy intake. Gut. (2020) 69:1258–68. 10.1136/gutjnl-2019-320438 32075887 PMC7306983

[92] Baxter NT Schmidt AW Venkataraman A Kim KS Waldron C Schmidt TM. Dynamics of human gut microbiota and short-chain fatty acids in response to dietary interventions with three fermentable fibers. mBio. (2019) 10:e02566-18. 10.1128/mBio.02566-18 30696735 PMC6355990

[93] Holmes ZC Villa MM Durand HK Jiang S Dallow EP Petrone BL. Microbiota responses to different prebiotics are conserved within individuals and associated with habitual fiber intake. Microbiome. (2022) 10:114. 10.1186/s40168-022-01307-x 35902900 PMC9336045

[94] Oliver A Chase AB Weihe C Orchanian SB Riedel SF Hendrickson CL. High-fiber, whole-food dietary intervention alters the human gut microbiome but not fecal short-chain fatty acids. mSystems. (2021) 6:e00115-21. 10.1128/mSystems.00115-21 33727392 PMC8546969

[95] Rodriguez-Garcia C Sanchez-Quesada C Algarra I Gaforio JJ. The high-fat diet based on extra-virgin olive oil causes dysbiosis linked to colorectal cancer prevention. Nutrients. (2020) 12:1705. 10.3390/nu12061705 32517306 PMC7352482

[96] Martin-Pelaez S Mosele JI Pizarro N Farras M de la Torre R Subirana I. Effect of virgin olive oil and thyme phenolic compounds on blood lipid profile: implications of human gut microbiota. Eur J Nutr. (2017) 56:119–31. 10.1007/s00394-015-1063-2 26541328

[97] Osborn LJ Schultz K Massey W DeLucia B Choucair I Varadharajan V. A gut microbial metabolite of dietary polyphenols reverses obesity-driven hepatic steatosis. Proc Natl Acad Sci USA. (2022) 119:e2202934119. 10.1073/pnas.2202934119 36417437 PMC9860326

[98] Palmas V Pisanu S Madau V Casula E Deledda A Cusano R. Gut microbiota markers associated with obesity and overweight in Italian adults. Sci Rep. (2021) 11:5532. 10.1038/s41598-021-84928-w 33750881 PMC7943584

[99] Di Rosa C Di Francesco L Spiezia C Khazrai YM. Effects of animal and vegetable proteins on gut microbiota in subjects with overweight or obesity. Nutrients. (2023) 15:2675. 10.3390/nu15122675 37375578 PMC10300930

[100] Pessoa J Belew GD Barroso C Egas C Jones JG. The gut microbiome responds progressively to fat and/or sugar-rich diets and is differentially modified by dietary fat and sugar. Nutrients. (2023) 15:2097. 10.3390/nu15092097 37432234 PMC10180990

[101] Ridaura VK Faith JJ Rey FE Cheng J Duncan AE Kau AL. Gut microbiota from twins discordant for obesity modulate metabolism in mice. Science. (2013) 341:1241214. 10.1126/science.1241214 24009397 PMC3829625

[102] Turnbaugh PJ Ley RE Mahowald MA Magrini V Mardis ER Gordon JI. An obesity-associated gut microbiome with increased capacity for energy harvest. Nature. (2006) 444:1027–31. 10.1038/nature05414 17183312

[103] Vrieze A Van Nood E Holleman F Salojarvi J Kootte RS Bartelsman JF. Transfer of intestinal microbiota from lean donors increases insulin sensitivity in individuals with metabolic syndrome. Gastroenterology. (2012) 143:913–6 e7. 10.1053/j.gastro.2012.06.031 22728514

[104] Allegretti JR Kassam Z Mullish BH Chiang A Carrellas M Hurtado J. Effects of fecal microbiota transplantation with oral capsules in obese patients. Clin Gastroenterol Hepatol. (2020) 18:855–63 e2. 10.1016/j.cgh.2019.07.006 31301451

[105] Bombin A Yan S Bombin S Mosley JD Ferguson JF. Obesity influences composition of salivary and fecal microbiota and impacts the interactions between bacterial taxa. Physiol Rep. (2022) 10:e15254. 10.14814/phy2.15254 35384379 PMC8980904

[106] Mishra SP Wang B Jain S Ding J Rejeski J Furdui CM. A mechanism by which gut microbiota elevates permeability and inflammation in obese/diabetic mice and human gut. Gut. (2023) 72:1848–65. 10.1136/gutjnl-2022-327365 36948576 PMC10512000

[107] Hersoug LG Moller P Loft S. Role of microbiota-derived lipopolysaccharide in adipose tissue inflammation, adipocyte size and pyroptosis during obesity. Nutr Res Rev. (2018) 31:153–63. 10.1017/S0954422417000269 29362018

[108] Krueger ES Lloyd TS Tessem JS. The accumulation and molecular effects of trimethylamine N-oxide on metabolic tissues: it's not all bad. Nutrients. (2021) 13:2873. 10.3390/nu13082873 34445033 PMC8400152

[109] Schugar RC Shih DM Warrier M Helsley RN Burrows A Ferguson D. The TMAO-producing enzyme flavin-containing monooxygenase 3 regulates obesity and the Beiging of white adipose tissue. Cell Rep. (2017) 19:2451–61. 10.1016/j.celrep.2017.06.053 28636934 PMC5672822

[110] Genser L Aguanno D Soula HA Dong L Trystram L Assmann K. Increased jejunal permeability in human obesity is revealed by a lipid challenge and is linked to inflammation and type 2 diabetes. J Pathol. (2018) 246:217–30. 10.1002/path.5134 29984492

[111] Ecklu-Mensah G Choo-Kang C Maseng MG Donato S Bovet P Viswanathan B. Gut microbiota and fecal short chain fatty acids differ with adiposity and country of origin: the METS-microbiome study. Nat Commun. (2023) 14:5160. 10.1038/s41467-023-40874-x 37620311 PMC10449869

[112] Ilyes T Silaghi CN Craciun AM. Diet-related changes of short-chain fatty acids in blood and feces in obesity and metabolic syndrome. Biology. (2022) 11:1556. 10.3390/biology11111556 36358258 PMC9687917

[113] Overby HB Ferguson JF. Gut microbiota-derived short-chain fatty acids facilitate microbiota:host cross talk and modulate obesity and hypertension. Curr Hypertens Rep. (2021) 23:8. 10.1007/s11906-020-01125-2 33537923 PMC7992370

[114] Yamamura R Nakamura K Ukawa S Okada E Nakagawa T Imae A. Fecal short-chain fatty acids and obesity in a community-based Japanese population: The DOSANCO Health Study. Obes Res Clin Pract. (2021) 15:345–50. 10.1016/j.orcp.2021.06.003 34127427

[115] Chambers ES Byrne CS Morrison DJ Murphy KG Preston T Tedford C. Dietary supplementation with inulin-propionate ester or inulin improves insulin sensitivity in adults with overweight and obesity with distinct effects on the gut microbiota, plasma metabolome and systemic inflammatory responses: a randomised cross-over trial. Gut. (2019) 68:1430–8. 10.1136/gutjnl-2019-318424 30971437 PMC6691855

[116] Igudesman D Crandell JL Corbin KD Hooper J Thomas JM Bulik CM. Associations of dietary intake with the intestinal microbiota and short-chain fatty acids among young adults with type 1 diabetes and overweight or obesity. J Nutr. (2023) 153:1178–88. 10.1016/j.tjnut.2022.12.017 36841667 PMC10356993

[117] Jamar G Santamarina AB Casagrande BP Estadella D de Rosso VV Wagner R. Prebiotic potencial of jucara berry on changes in gut bacteria and acetate of individuals with obesity. Eur J Nutr. (2020) 59:3767–78. 10.1007/s00394-020-02208-1 32108262

[118] Gyarmati P Song Y Dotimas J Yoshiba G Christison A. Cross-sectional comparisons of gut microbiome and short-chain fatty acid levels among children with varied weight classifications. Pediatr Obes. (2021) 16:e12750. 10.1111/ijpo.12750 33174684

[119] Wei Y Liang J Su Y Wang J Amakye WK Pan J. The associations of the gut microbiome composition and short-chain fatty acid concentrations with body fat distribution in children. Clin Nutr. (2021) 40:3379–90. 10.1016/j.clnu.2020.11.014 33277072

[120] Slizewska K Wlodarczyk M Sobczak M Barczynska R Kapusniak J Socha P. Comparison of the activity of fecal enzymes and concentration of SCFA in healthy and overweight children. Nutrients. (2023) 15:987. 10.3390/nu15040987 36839343 PMC9966664

[121] Andre P Pais de Barros JP Mj Merle B Samieri C Helmer C Delcourt C. Mediterranean diet and prudent diet are both associated with low circulating esterified 3-hydroxy fatty acids, a proxy of LPS burden, among older adults. Am J Clin Nutr. (2021) 114:1080–91. 10.1093/ajcn/nqab126 34036325

[122] Martinez-Gonzalez MA Fernandez-Jarne E Serrano-Martinez M Wright M Gomez-Gracia E. Development of a short dietary intake questionnaire for the quantitative estimation of adherence to a cardioprotective Mediterranean diet. Eur J Clin Nutr. (2004) 58:1550–2. 10.1038/sj.ejcn.1602004 15162136

[123] Barrea L Annunziata G Muscogiuri G Laudisio D Di Somma C Maisto M. Trimethylamine N-oxide, Mediterranean diet, and nutrition in healthy, normal-weight adults: also a matter of sex? Nutrition. (2019) 62:7–17. 10.1016/j.nut.2018.11.015 30822745

[124] Martinez-Gonzalez MA Corella D Salas-Salvado J Ros E Covas MI Fiol M. Cohort profile: design and methods of the PREDIMED study. Int J Epidemiol. (2012) 41:377–85. 10.1093/ije/dyq250 21172932

[125] Barrea L Muscogiuri G Pugliese G Graziadio C Maisto M Pivari F. Association of the chronotype score with circulating trimethylamine N-oxide (TMAO) concentrations. Nutrients. (2021) 13:1671. 10.3390/nu13051671 34069075 PMC8156852

[126] Martinez-Gonzalez MA Garcia-Arellano A Toledo E Salas-Salvado J Buil-Cosiales P Corella D. A 14-item mediterranean diet assessment tool and obesity indexes among high-risk subjects: the PREDIMED trial. PLoS ONE. (2012) 7:e0043134. 10.1371/journal.pone.0043134 22905215 PMC3419206

[127] Barrea L Muscogiuri G Pugliese G de Alteriis G Maisto M Donnarumma M. Association of trimethylamine N-Oxide (TMAO) with the clinical severity of hidradenitis suppurativa (Acne Inversa). Nutrients. (2021) 13:1997. 10.3390/nu13061997 34200594 PMC8226830

[128] De Filippis F Pellegrini N Vannini L Jeffery IB La Storia A Laghi L. High-level adherence to a Mediterranean diet beneficially impacts the gut microbiota and associated metabolome. Gut. (2016) 65:1812–21. 10.1136/gutjnl-2015-309957 26416813

[129] Agnoli C Krogh V Grioni S Sieri S Palli D Masala G. A priori-defined dietary patterns are associated with reduced risk of stroke in a large Italian cohort. J Nutr. (2011) 141:1552–8. 10.3945/jn.111.140061 21628636

[130] Galie S Garcia-Gavilan J Papandreou C Camacho-Barcia L Arcelin P Palau-Galindo A. Effects of Mediterranean Diet on plasma metabolites and their relationship with insulin resistance and gut microbiota composition in a crossover randomized clinical trial. Clin Nutr. (2021) 40:3798–806. 10.1016/j.clnu.2021.04.028 34130026

[131] Martinez-Gonzalez MA Buil-Cosiales P Corella D Bullo M Fito M Vioque J. Cohort profile: design and methods of the PREDIMED-Plus randomized trial. Int J Epidemiol. (2019) 48:387–8o. 10.1093/ije/dyy225 30476123

[132] Garcia-Mantrana I Selma-Royo M Alcantara C Collado MC. Shifts on gut microbiota associated to mediterranean diet adherence and specific dietary intakes on general adult population. Front Microbiol. (2018) 9:890. 10.3389/fmicb.2018.00890 29867803 PMC5949328

[133] Berendsen AAM van de Rest O Feskens EJM Santoro A Ostan R Pietruszka B. Changes in dietary intake and adherence to the NU-AGE diet following a one-year dietary intervention among european older adults-results of the NU-AGE randomized trial. Nutrients. (2018) 10:1905. 10.3390/nu10121905 30518044 PMC6315357

[134] Griffin LE Djuric Z Angiletta CJ Mitchell CM Baugh ME Davy KP. Mediterranean diet does not alter plasma trimethylamine N-oxide concentrations in healthy adults at risk for colon cancer. Food Funct. (2019) 10:2138–47. 10.1039/C9FO00333A 30938383 PMC6552673

[135] Sidahmed E Cornellier ML Ren J Askew LM Li Y Talaat N. Development of exchange lists for Mediterranean and Healthy Eating diets: implementation in an intervention trial. J Hum Nutr Diet. (2014) 27:413–25. 10.1111/jhn.12158 24112099 PMC3961569

[136] Guasch-Ferre M Hu FB Ruiz-Canela M Bullo M Toledo E Wang DD. Plasma metabolites from choline pathway and risk of cardiovascular disease in the PREDIMED (prevention with mediterranean diet) Study. J Am Heart Assoc. (2017) 6:6524. 10.1161/JAHA.117.006524 29080862 PMC5721752

[137] Gutierrez-Diaz I Fernandez-Navarro T Sanchez B Margolles A Gonzalez S. Mediterranean diet and faecal microbiota: a transversal study. Food Funct. (2016) 7:2347–56. 10.1039/C6FO00105J 27137178

[138] Gonzalez S Fernandez M Cuervo A Lasheras C. Dietary intake of polyphenols and major food sources in an institutionalised elderly population. J Hum Nutr Diet. (2014) 27:176–83. 10.1111/jhn.12058 23521491

[139] Trichopoulou A Kouris-Blazos A Wahlqvist ML Gnardellis C Lagiou P Polychronopoulos E. Diet and overall survival in elderly people. BMJ. (1995) 311:1457–60. 10.1136/bmj.311.7018.1457 8520331 PMC2543726

[140] Monteagudo C Mariscal-Arcas M Rivas A Lorenzo-Tovar ML Tur JA Olea-Serrano F. Proposal of a Mediterranean Diet Serving Score. PLoS ONE. (2015) 10:e0128594. 10.1371/journal.pone.0128594 26035442 PMC4452755

[141] Krishnan S O'Connor LE Wang Y Gertz ER Campbell WW Bennett BJ. Adopting a Mediterranean-style eating pattern with low, but not moderate, unprocessed, lean red meat intake reduces fasting serum trimethylamine N-oxide (TMAO) in adults who are overweight or obese. Br J Nutr. (2021) 128:1–21. 10.1017/S0007114521004694 34823615 PMC9133270

[142] Maldonado-Contreras A Noel SE Ward DV Velez M Mangano KM. Associations between diet, the gut microbiome, and short-chain fatty acid production among older Caribbean Latino adults. J Acad Nutr Diet. (2020) 120:2047–60 e6. 10.1016/j.jand.2020.04.018 32798072

[143] Trichopoulou A Costacou T Bamia C Trichopoulos D. Adherence to a Mediterranean diet and survival in a Greek population. N Engl J Med. (2003) 348:2599–608. 10.1056/NEJMoa025039 12826634

[144] Mitsou EK Kakali A Antonopoulou S Mountzouris KC Yannakoulia M Panagiotakos DB. Adherence to the Mediterranean diet is associated with the gut microbiota pattern and gastrointestinal characteristics in an adult population. Br J Nutr. (2017) 117:1645–55. 10.1017/S0007114517001593 28789729

[145] Panagiotakos DB Pitsavos C Stefanadis C. Dietary patterns: a Mediterranean diet score and its relation to clinical and biological markers of cardiovascular disease risk. Nutr Metab Cardiovasc Dis. (2006) 16:559–68. 10.1016/j.numecd.2005.08.006 17126772

[146] Nagpal R Neth BJ Wang S Craft S Yadav H. Modified Mediterranean-ketogenic diet modulates gut microbiome and short-chain fatty acids in association with Alzheimer's disease markers in subjects with mild cognitive impairment. EBioMedicine. (2019) 47:529–42. 10.1016/j.ebiom.2019.08.032 31477562 PMC6796564

[147] Sofi F Macchi C Abbate R Gensini GF Casini A. Mediterranean diet and health status: an updated meta-analysis and a proposal for a literature-based adherence score. Public Health Nutr. (2014) 17:2769–82. 10.1017/S1368980013003169 24476641 PMC10282340

[148] Park JE Miller M Rhyne J Wang Z Hazen SL. Differential effect of short-term popular diets on TMAO and other cardio-metabolic risk markers. Nutr Metab Cardiovasc Dis. (2019) 29:513–7. 10.1016/j.numecd.2019.02.003 30940489

[149] Pastori D Carnevale R Nocella C Novo M Santulli M Cammisotto V. Gut-derived serum lipopolysaccharide is associated with enhanced risk of major adverse cardiovascular events in atrial fibrillation: effect of adherence to Mediterranean diet. J Am Heart Assoc. (2017) 6:5784. 10.1161/JAHA.117.005784 28584074 PMC5669181

[150] Pastori D Ettorre E Carnevale R Nocella C Bartimoccia S Del Sordo E. Interaction between serum endotoxemia and proprotein convertase subtilisin/kexin 9 (PCSK9) in patients with atrial fibrillation: a *post-hoc* analysis from the ATHERO-AF cohort. Atherosclerosis. (2019) 289:195–200. 10.1016/j.atherosclerosis.2019.07.002 31303312

[151] Pignanelli M Just C Bogiatzi C Dinculescu V Gloor GB Allen-Vercoe E. Mediterranean diet score: associations with metabolic products of the intestinal microbiome, carotid plaque burden, and renal function. Nutrients. (2018) 10:779. 10.3390/nu10060779 29914158 PMC6024790

[152] Fung TT Rexrode KM Mantzoros CS Manson JE Willett WC Hu FB. Mediterranean diet and incidence of and mortality from coronary heart disease and stroke in women. Circulation. (2009) 119:1093–100. 10.1161/CIRCULATIONAHA.108.816736 19221219 PMC2724471

[153] Quercia S Turroni S Fiori J Soverini M Rampelli S Biagi E. Gut microbiome response to short-term dietary interventions in reactive hypoglycemia subjects. Diabetes Metab Res Rev. (2017) 33:2927. 10.1002/dmrr.2927 28806487

[154] Ruiz-Saavedra S Salazar N Suarez A de Los Reyes-Gavilan CG Gueimonde M Gonzalez S. Comparison of different dietary indices as predictors of inflammation, oxidative stress and intestinal microbiota in middle-aged and elderly subjects. Nutrients. (2020) 12:3828. 10.3390/nu12123828 33333806 PMC7765160

[155] Mariscal-Arcas M Romaguera D Rivas A Feriche B Pons A Tur JA. Diet quality of young people in southern Spain evaluated by a Mediterranean adaptation of the Diet Quality Index-International (DQI-I). Br J Nutr. (2007) 98:1267–73. 10.1017/S0007114507781424 17640424

[156] Buckland G Agudo A Travier N Huerta JM Cirera L Tormo MJ. Adherence to the Mediterranean diet reduces mortality in the Spanish cohort of the European Prospective Investigation into Cancer and Nutrition (EPIC-Spain). Br J Nutr. (2011) 106:1581–91. 10.1017/S0007114511002078 21736834

[157] Trichopoulou A Orfanos P Norat T Bueno-de-Mesquita B Ocke MC Peeters PH. Modified Mediterranean diet and survival: EPIC-elderly prospective cohort study. BMJ. (2005) 330:991. 10.1136/bmj.38415.644155.8F 15820966 PMC557144

[158] Seethaler B Nguyen NK Basrai M Kiechle M Walter J Delzenne NM. Short-chain fatty acids are key mediators of the favorable effects of the Mediterranean diet on intestinal barrier integrity: data from the randomized controlled LIBRE trial. Am J Clin Nutr. (2022) 116:928–42. 10.1093/ajcn/nqac175 36055959

[159] Schroder H Fito M Estruch R Martinez-Gonzalez MA Corella D Salas-Salvado J. A short screener is valid for assessing mediterranean diet adherence among older Spanish men and women. J. Nutr. (2011) 141:1140–5. 10.3945/jn.110.135566 21508208

[160] Hebestreit K Yahiaoui-Doktor M Engel C Vetter W Siniatchkin M Erickson N. Validation of the German version of the Mediterranean Diet Adherence Screener (MEDAS) questionnaire. BMC Cancer. (2017) 17:341. 10.1186/s12885-017-3337-y 28521737 PMC5437541

[161] Seethaler B Lehnert K Yahiaoui-Doktor M Basrai M Vetter W Kiechle M. Omega-3 polyunsaturated fatty acids improve intestinal barrier integrity-albeit to a lesser degree than short-chain fatty acids: an exploratory analysis of the randomized controlled LIBRE trial. Eur J Nutr. (2023) 62:2779–91. 10.1007/s00394-023-03172-2 37318580 PMC10468946

[162] Shankar V Gouda M Moncivaiz J Gordon A Reo NV Hussein L Paliy O. Differences in gut metabolites and microbial composition and functions between Egyptian and U.S. children are consistent with their diets. mSystems. (2017) 62:2779–91. 10.1128/mSystems.00169-16 28191503 PMC5296411

[163] Shoer S Shilo S Godneva A Ben-Yacov O Rein M Wolf BC. Impact of dietary interventions on pre-diabetic oral and gut microbiome, metabolites and cytokines. Nat Commun. (2023) 14:5384. 10.1038/s41467-023-41042-x 37666816 PMC10477304

[164] Papadaki A Johnson L Toumpakari Z England C Rai M Toms S. Validation of the English Version of the 14-Item Mediterranean diet adherence screener of the PREDIMED study, in people at high cardiovascular risk in the UK. Nutrients. (2018) 10:138. 10.3390/nu10020138 29382082 PMC5852714

[165] Tanaka T Talegawkar SA Jin Y Candia J Tian Q Moaddel R. Metabolomic profile of different dietary patterns and their association with frailty index in community-dwelling older men and women. Nutrients. (2022) 14:2237. 10.3390/nu14112237 35684039 PMC9182888

[166] Morris MC Tangney CC Wang Y Sacks FM Barnes LL Bennett DA. MIND diet slows cognitive decline with aging. Alzheimers Dement. (2015) 11:1015–22. 10.1016/j.jalz.2015.04.011 26086182 PMC4581900

[167] Vitale M Giacco R Laiola M Della Pepa G Luongo D Mangione A. Acute and chronic improvement in postprandial glucose metabolism by a diet resembling the traditional Mediterranean dietary pattern: Can SCFAs play a role? Clin Nutr. (2021) 40:428–37. 10.1016/j.clnu.2020.05.025 32698959

[168] Zhu CH Sawrey-Kubicek L Beals E Rhodes CH Houts HE Sacchi R. Human gut microbiome composition and tryptophan metabolites were changed differently by fast food and Mediterranean diet in 4 days: a pilot study. Nutrition Research. (2020) 77:62–72. 10.1016/j.nutres.2020.03.005 32330749

[169] Hernandez MAG Canfora EE Jocken JWE Blaak EE. The short-chain fatty acid acetate in body weight control and insulin sensitivity. Nutrients. (2019) 11:1943. 10.3390/nu11081943 31426593 PMC6723943

[170] Arifuzzaman M Collins N Guo CJ Artis D. Nutritional regulation of microbiota-derived metabolites: Implications for immunity and inflammation. Immunity. (2024) 57:14–27. 10.1016/j.immuni.2023.12.009 38198849 PMC10795735

[171] Bachem A Makhlouf C Binger KJ de Souza DP Tull D Hochheiser K. Microbiota-derived short-chain fatty acids promote the memory potential of antigen-activated CD8(+) T cells. Immunity. (2019) 51:285–97 e5. 10.1016/j.immuni.2019.06.002 31272808

[172] Hang S Paik D Yao L Kim E Trinath J Lu J. Bile acid metabolites control T(H)17 and T(reg) cell differentiation. Nature. (2019) 576:143–8. 10.1038/s41586-019-1785-z 31776512 PMC6949019

[173] Paik D Yao L Zhang Y Bae S D'Agostino GD Zhang M. Human gut bacteria produce Tau(Eta)17-modulating bile acid metabolites. Nature. (2022) 603:907–12. 10.1038/s41586-022-04480-z 35296854 PMC9132548

[174] Arifuzzaman M Won TH Li TT Yano H Digumarthi S Heras AF. Inulin fibre promotes microbiota-derived bile acids and type 2 inflammation. Nature. (2022) 611:578–84. 10.1038/s41586-022-05380-y 36323778 PMC10576985

[175] Hoyles L Pontifex MG Rodriguez-Ramiro I Anis-Alavi MA Jelane KS Snelling T. Regulation of blood brain barrier integrity by microbiome-associated methylamines and cognition by trimethylamine N-oxide. Microbiome. (2021) 9:235. 10.1186/s40168-021-01181-z 34836554 PMC8626999

[176] Islam T Albracht-Schulte K Ramalingam L Schlabritz-Lutsevich N Park OH Zabet-Moghaddam M. Anti-inflammatory mechanisms of polyphenols in adipose tissue: role of gut microbiota, intestinal barrier integrity and zinc homeostasis. J Nutr Biochem. (2023) 115:109242. 10.1016/j.jnutbio.2022.109242 36442715

[177] Vicentini FA Keenan CM Wallace LE Woods C Cavin JB Flockton AR. Intestinal microbiota shapes gut physiology and regulates enteric neurons and glia. Microbiome. (2021) 9:210. 10.1186/s40168-021-01165-z 34702353 PMC8549243

[178] Shi H Ge X Ma X Zheng M Cui X Pan W. A fiber-deprived diet causes cognitive impairment and hippocampal microglia-mediated synaptic loss through the gut microbiota and metabolites. Microbiome. (2021) 9:223. 10.1186/s40168-021-01172-0 34758889 PMC8582174

[179] Gutierrez-Repiso C Molina-Vega M Bernal-Lopez MR Garrido-Sanchez L Garcia-Almeida JM Sajoux I. Different weight loss intervention approaches reveal a lack of a common pattern of gut microbiota changes. J Pers Med. (2021) 11:109. 10.3390/jpm11020109 33567649 PMC7915884

[180] Lane MM Davis JA Beattie S Gomez-Donoso C Loughman A O'Neil A. Ultraprocessed food and chronic noncommunicable diseases: A systematic review and meta-analysis of 43 observational studies. Obes Rev. (2021) 22:e13146. 10.1111/obr.13146 33167080

[181] Kapoor P Tiwari A Sharma S Tiwari V Sheoran B Ali U Garg M. Effect of anthocyanins on gut health markers, Firmicutes-Bacteroidetes ratio and short-chain fatty acids: a systematic review via meta-analysis. Sci. Rep. (2023) 13:1729. 10.1038/s41598-023-28764-0 36720989 PMC9889808

[182] Sowah SA Riedl L Damms-Machado A Johnson TS Schubel R Graf M. Effects of weight-loss interventions on short-chain fatty acid concentrations in blood and feces of adults: a systematic review. Adv Nutr. (2019) 10:673–84. 10.1093/advances/nmy125 31075175 PMC6628843

[183] Farhat Z Sampson JN Hildesheim A Safaeian M Porras C Cortes B. Reproducibility, temporal variability, and concordance of serum and fecal bile acids and short chain fatty acids in a population-based study. Cancer Epidemiol Biomarkers Prev. (2021) 30:1875–83. 10.1158/1055-9965.EPI-21-0361 34376486 PMC8608567

[184] Bailey MA Holscher HD. Microbiome-mediated effects of the mediterranean diet on inflammation. Adv Nutr. (2018) 9:193–206. 10.1093/advances/nmy013 29767701 PMC5952955

[185] Hu Y Chen Z Xu C Kan S Chen D. Disturbances of the gut microbiota and microbiota-derived metabolites in inflammatory Bowel disease. Nutrients. (2022) 14:5140. 10.3390/nu14235140 36501169 PMC9735443

[186] Quinn RA Melnik AV Vrbanac A Fu T Patras KA Christy MP. Global chemical effects of the microbiome include new bile-acid conjugations. Nature. (2020) 579:123–9. 10.1038/s41586-020-2047-9 32103176 PMC7252668

[187] Bustamante JM Dawson T Loeffler C Marfori Z Marchesi JR Mullish BH. Impact of fecal microbiota transplantation on gut bacterial bile acid metabolism in humans. Nutrients. (2022) 14:5200. 10.3390/nu14245200 36558359 PMC9785599

[188] Li M Wang S Li Y Zhao M Kuang J Liang D. Gut microbiota-bile acid crosstalk contributes to the rebound weight gain after calorie restriction in mice. Nat Commun. (2022) 13:2060. 10.1038/s41467-022-29589-7 35440584 PMC9018700

[189] von Schwartzenberg RJ Bisanz JE Lyalina S Spanogiannopoulos P Ang QY Cai J. Caloric restriction disrupts the microbiota and colonization resistance. Nature. (2021) 595:272–7. 10.1038/s41586-021-03663-4 34163067 PMC8959578

[190] Xiao C Wang JT Su C Miao Z Tang J Ouyang Y. Associations of dietary diversity with the gut microbiome, fecal metabolites, and host metabolism: results from 2 prospective Chinese cohorts. Am J Clin Nutr. (2022) 116:1049–58. 10.1093/ajcn/nqac178 36100971 PMC9535526

[191] Seconda L Baudry J Alles B Hamza O Boizot-Szantai C Soler LG. Assessment of the sustainability of the Mediterranean diet combined with organic food consumption: an individual behaviour approach. Nutrients. (2017) 9:61. 10.3390/nu9010061 28085096 PMC5295105

[192] Newman NK Zhang Y Padiadpu J Miranda CL Magana AA Wong CP. Reducing gut microbiome-driven adipose tissue inflammation alleviates metabolic syndrome. Microbiome. (2023) 11:208. 10.1186/s40168-023-01637-4 37735685 PMC10512512

[193] Faits T Walker ME Rodriguez-Morato J Meng H Gervis JE Galluccio JM. Exploring changes in the human gut microbiota and microbial-derived metabolites in response to diets enriched in simple, refined, or unrefined carbohydrate-containing foods: a post hoc analysis of a randomized clinical trial. Am J Clin Nutr. (2020) 112:1631–41. 10.1093/ajcn/nqaa254 32936872 PMC7727488

[194] Janeiro MH Ramirez MJ Milagro FI Martinez JA Solas M. Implication of trimethylamine N-oxide (TMAO) in disease: potential biomarker or new therapeutic target. Nutrients. (2018) 10:1398. 10.3390/nu10101398 30275434 PMC6213249

[195] Chen K Zheng X Feng M Li D Zhang H. Gut microbiota-dependent metabolite trimethylamine N-oxide contributes to cardiac dysfunction in western diet-induced obese mice. Front Physiol. (2017) 8:139. 10.3389/fphys.2017.00139 28377725 PMC5359299

[196] Papandreou C Bullo M Zheng Y Ruiz-Canela M Yu E Guasch-Ferre M. Plasma trimethylamine-N-oxide and related metabolites are associated with type 2 diabetes risk in the Prevencion con Dieta Mediterranea (PREDIMED) trial. Am J Clin Nutr. (2018) 108:163–73. 10.1093/ajcn/nqy058 29982310 PMC6862602

[197] Diez-Ricote L San-Cristobal R Concejo MJ Martinez-Gonzalez MA Corella D Salas-Salvado J. One-year longitudinal association between changes in dietary choline or betaine intake and cardiometabolic variables in the PREvencion con DIeta MEDiterranea-Plus (PREDIMED-Plus) trial. Am J Clin Nutr. (2022) 116:1565–79. 10.1093/ajcn/nqac255 36124652 PMC9761742

[198] Jang H Lim H Park KH Park S Lee HJ. Changes in plasma choline and the betaine-to-choline ratio in response to 6-month lifestyle intervention are associated with the changes of lipid profiles and intestinal microbiota: The ICAAN Study. Nutrients. (2021) 13:6. 10.3390/nu13114006 34836260 PMC8625635

[199] Ghanim H Batra M Abuaysheh S Green K Makdissi A Kuhadiya ND. Antiinflammatory and ROS suppressive effects of the addition of fiber to a high-fat high-calorie meal. J Clin Endocrinol Metab. (2017) 102:858–69. 10.1210/jc.2016-2669 27906549

[200] Pendyala S Walker JM Holt PR. A high-fat diet is associated with endotoxemia that originates from the gut. Gastroenterology. (2012) 142:1100–1 e2. 10.1053/j.gastro.2012.01.034 22326433 PMC3978718

[201] Hasegawa Y Pei R Raghuvanshi R Liu Z Bolling BW. Yogurt supplementation attenuates insulin resistance in obese mice by reducing metabolic endotoxemia and inflammation. J Nutr. (2023) 153:703–12. 10.1016/j.tjnut.2023.01.021 36774230

[202] Johnson SL Kirk RD DaSilva NA Ma H Seeram NP Bertin MJ. Polyphenol microbial metabolites exhibit gut and blood(-)brain barrier permeability and protect murine microglia against LPS-induced inflammation. Metabolites. (2019) 9:78. 10.3390/metabo9040078 31010159 PMC6523162

[203] Bartimoccia S Cammisotto V Nocella C Del Ben M D'Amico A Castellani V. Extra virgin olive oil reduces gut permeability and metabolic endotoxemia in diabetic patients. Nutrients. (2022) 14:2153. 10.3390/nu14102153 35631294 PMC9145083

[204] Gundogdu A Nalbantoglu OU. The role of the Mediterranean diet in modulating the gut microbiome: a review of current evidence. Nutrition. (2023) 114:112118. 10.1016/j.nut.2023.112118 37437419

[205] Fassarella M Blaak EE Penders J Nauta A Smidt H Zoetendal EG. Gut microbiome stability and resilience: elucidating the response to perturbations in order to modulate gut health. Gut. (2021) 70:595–605. 10.1136/gutjnl-2020-321747 33051190

